# Epstein-Barr Virus BGLF2 commandeers RISC to interfere with cellular miRNA function

**DOI:** 10.1371/journal.ppat.1010235

**Published:** 2022-01-10

**Authors:** Ashley M. Campbell, Carlos F. De La Cruz-Herrera, Edyta Marcon, Jack Greenblatt, Lori Frappier

**Affiliations:** 1 Department of Molecular Genetics, University of Toronto, Toronto, Canada; 2 Donnelly Centre, University of Toronto, Toronto, Canada; Duke University Medical Center, UNITED STATES

## Abstract

The Epstein-Barr virus (EBV) BGLF2 protein is a tegument protein with multiple effects on the cellular environment, including induction of SUMOylation of cellular proteins. Using affinity-purification coupled to mass-spectrometry, we identified the miRNA-Induced Silencing Complex (RISC), essential for miRNA function, as a top interactor of BGLF2. We confirmed BGLF2 interaction with the Ago2 and TNRC6 components of RISC in multiple cell lines and their co-localization in cytoplasmic bodies that also contain the stress granule marker G3BP1. In addition, BGLF2 expression led to the loss of processing bodies in multiple cell types, suggesting disruption of RISC function in mRNA regulation. Consistent with this observation, BGLF2 disrupted Ago2 association with multiple miRNAs. Using let-7 miRNAs as a model, we tested the hypothesis that BGLF2 interfered with the function of RISC in miRNA-mediated mRNA silencing. Using multiple reporter constructs with 3’UTRs containing let-7a regulated sites, we showed that BGLF2 inhibited let-7a miRNA activity dependent on these 3’UTRs, including those from SUMO transcripts which are known to be regulated by let-7 miRNAs. In keeping with these results, we showed that BGLF2 increased the cellular level of unconjugated SUMO proteins without affecting the level of SUMO transcripts. Such an increase in free SUMO is known to drive SUMOylation and would account for the effect of BGLF2 in inducing SUMOylation. We further showed that BGLF2 expression inhibited the loading of let-7 miRNAs into Ago2 proteins, and conversely, that lytic infection with EBV lacking BGLF2 resulted in increased interaction of let-7a and SUMO transcripts with Ago2, relative to WT EBV infection. Therefore, we have identified a novel role for BGLF2 as a miRNA regulator and shown that one outcome of this activity is the dysregulation of SUMO transcripts that leads to increased levels of free SUMO proteins and SUMOylation.

## Author summary

Epstein-Barr virus (EBV) infects most people worldwide, persists for life and is associated with several kinds of cancer. In order to undergo efficient lytic infection, EBV must manipulate multiple cellular pathways. BGLF2 is an EBV lytic protein known to modulate several cellular processes including increasing the modification of cellular proteins with the Small Ubiquitin-Like Modifier (SUMO), a process referred to as SUMOylation. Here we show for the first time that BGLF2 interacts with a cellular complex (RISC) required for miRNA function and interferes with the function of some cellular miRNAs by sequestering this complex. One of the consequences of this effect is the increased expression of SUMO proteins, due to inhibition of the miRNAs that normally downregulate their expression. The resulting increase in SUMO proteins drives SUMOylation, providing a mechanism for the previously reported BGLF2-induced SUMOylation of cellular proteins. In addition, the discovery of BGLF2 as a miRNA regulator suggests that this EBV protein can control many cellular pathways by interfering with cellular miRNAs that normally regulate them.

## Introduction

Epstein-Barr virus (EBV) is a γ-herpesvirus that infects ~90% of the global population and is a causative agent for several kinds of lymphomas, nasopharyngeal carcinoma, and ~10% of gastric carcinomas. Like all herpesviruses, EBV can alternate between latent and lytic forms of infection. Latent infection involves the expression of a small subset of EBV proteins, cell immortalization and maintenance of EBV episomes at a constant copy number. Lytic infection involves expression of an ordered cascade of ~80 proteins, EBV genome amplification and production of virions. In addition, EBV encodes 25 primary microRNAs (pri-miRNAs) which produce 44 mature miRNAs [1,2]. These miRNAs, which have been mainly studied in the context of EBV-induced cancers, regulate the functions of both cellular and viral mRNAs, at least in part to inhibit innate and adaptive immune responses (reviewed in [3–5]).

miRNAs are evolutionarily conserved across eukaryotes as small non-coding RNAs (21–25 nts) important for mRNA regulation [6,7]. Cellular- and EBV-derived pri-miRNAs are cleaved by DROSHA/DGCR8 to produce precursor miRNAs (pre-miRNAs) [8,9]. Pre-miRNAs are then exported from the nucleus to the cytoplasm by exportin 5 (XPO5; [10–12]). Cytoplasmic pre-miRNAs are further processed by Dicer to produce a mature ~22-nucleotide miRNA duplexes. Dicer associates with the transactivation-responsive RNA-binding protein (TRBP; also called TARBP2) which bridges the interaction between Dicer and Argonaute (Ago1, -2, -3, or -4) proteins [13]. Argonaute is the main component of the microribonucleoprotein complex called the miRNA-Induced Silencing Complex (RISC or miRISC), which also contains TNRC6 proteins. TNRC6A (also called GW182), TNRC6B, and TNRC6C are functionally redundant [14–17], and all can interact any Ago protein to form different versions of RISC. One strand of the miRNA duplex (guide strand), bound by Dicer, is loaded into an Ago protein, and RISC is recruited to targeted mRNAs by the partial complementarity of the miRNAs loaded into Ago [14,18,19]. Ago2 is the only human Argonaute protein with an endonuclease activity that cleaves mRNA when a small non-coding RNA has complete complementarity to the target mRNA [20,21]. miRNA-mediated degradation is also promoted by the TNRC6 component of RISC, which recruits the CCR4-NOT deadenylation complex and the decapping activator DDX6 [14]. TNRC6 also recruits eIF4E2 to inhibit translation initiation [14,18].

Herpesviruses express a large number of proteins (~80–200) in lytic infection, many of which are known or likely to function to alter the cellular environment to promote infection. Accumulating studies indicate that even proteins with well characterized direct roles in viral infection have additional role in manipulating cellular processes [22–25], although many have yet to be investigated. We have previously screened a library of EBV proteins for cellular changes associated with EBV lytic infection, including disruption of PML nuclear bodies [26], induction of G_1_/S arrest [27], inhibition of the DNA damage response [28] and modulation of cellular SUMOylation [23]. The EBV protein BGLF2 was found to promote G_1_/S cell cycle arrest and to induce global SUMOylation of cellular proteins, indicating that it has some capacity to modulate cellular processes [23,27].

BGLF2 is an EBV tegument protein that is expressed late in EBV lytic infection [29]. BGLF2 interacts with EBV tegument components BBLF1 and BKRF4 and is required for efficient production of virions [29–31]. In addition, BGLF2 was found to activate AP-1 promoters, leading to upregulation of the mitogen-activated protein kinase (MAPK) pathway and increased transcription of the EBV BZLF1 lytic switch gene [31,32]. BGLF2 has also been reported to inhibit interferon (IFN) signaling and NF-κB activity by preventing p65 phosphorylation [32–35]. In addition to these roles in inhibiting immune signaling, we had previously found that BGLF2 upregulated p21 levels and promoted G_1_/S cell cycle arrest through interactions with the cellular NIMA-related protein kinase (HNEK9) and GEM-interacting protein (GMIP) [27]. Finally, BGLF2 was found to globally upregulate the SUMOylation of cellular proteins by an unknown mechanism [23]. The modification of proteins by the addition of the Small Ubiquitin-like Modifiers (SUMOs) is an important mechanism of regulating both viral and cellular proteins that can affect their activity, localization, or stability, and can promote protein-protein interactions. SUMOylation of cellular proteins is often associated with antiviral and stress responses and viruses are known to encode proteins that induce SUMOylation or target SUMOylated cellular proteins for degradation [23,36–41].

To better understand the mechanisms of action of BGLF2, we used affinity-purification coupled to mass-spectrometry (AP-MS) to profile the host protein interactions of BGLF2. This revealed a previously unknown interaction with the RISC, a miRNA-mediated regulatory complex, implicating BGLF2 as a miRNA regulator. Here, we show that BGLF2 interferes with let-7 miRNAs function, including its role in regulating the expression of SUMO proteins. As a result, BGLF2 increases free SUMO levels and thereby drives SUMOylation, providing a mechanism for the induction of SUMOylation previously observed for BGLF2.

## Results

### BGLF2 interacts with components of RISC

To better understand the cellular effects of BGLF2, we performed affinity purification-mass spectrometry (AP-MS) with BGLF2 to identify the cellular proteins it targets. To this end, FLAG-tagged BGLF2 was expressed in 293T cells, recovered on anti-FLAG resin, and co-purifying proteins were trypsinized and analyzed by liquid chromatography-tandem mass spectroscopy (LC-MS/MS). Peptide recovery (“spectral counts”) from two independent experiments with BGLF2 were compared to empty FLAG plasmid controls and to the average peptide recovery in the Contaminant Repository for Affinity Purification (CRAPome), a database of peptide recovery in over 400 AP-MS experiments [42] to identify nonspecific interactors. The top 30 interactors that were obtained in both experiments, but not in the empty plasmid control, and at levels significantly higher than the CRAPome, are shown in Table 1. Among the top interactors was the previously characterized interaction with HNEK9 that is associated with p21 induction by BGLF2 [27]. In addition, prominent interactions were discovered for proteins and complexes important for RNA regulation. These proteins include eIF4E2 (also called 4EHP), GIGYF1/2, and ZNF598, which form complexes that function in translation silencing and ribosome-associated quality control (RQC; [43–46]). Other prominent interacting RNA regulatory proteins include all of the trinucleotide repeat containing 6 (TNRC6) paralogs (A, B, C), as well as Argonaute (Ago) proteins 1 and 2, which are the main protein components of the miRNA-Induced Silencing Complex (RISC). RISC is typically found in processing-bodies (p-bodies), where it functions in miRNA regulation and, under stress conditions, also localizes with stalled mRNA-protein complexes in stress granules. In keeping with the BGLF2-RISC interaction, several proteins recovered with BGLF2 were components of stress granules and p-bodies. These BGLF2 interactions suggests a role for BGLF2 in miRNA regulation.

**Table 1:** Affinity Purification-Mass Spectrometry Performed with BGLF2 Reveals an Interaction with RISC.

		Total Spectral Counts				
Uniprot ID	Protein	Empty FLAG plasmid	BGLF2-FLAG Exp1|Exp2	CRAPome Average	Avg SC/ a.a.*	Localizes to stress granules (SG) or P- bodies (PB)
P0CK53	EBV BGLF2	0	181|234	N/A	0.61756	
Q6Y7W6	GIGYF2	0	167|147	20.5	0.12086	SG [47,48]
Q5JSZ5	PRRC2B	0	131|73	2	0.04576	SG [47]
Q9UPQ9^t^	TNRC6B	0	114|150	3	0.07201	SG and PB [47]
Q9Y520	PRRC2C	0	95|51	10.1	0.02521	SG [47,49]
Q8N6T3	ARFGAP1	0	74|86	6.4	0.19704	
Q14157	UBAP2L	0	71|81	10.4	0.06992	SG [47,49]
O60573	EIF4E2	0	71|59	8.6	0.26531	SG and PB [47]
Q14674	ESPL1	0	60|44	1	0.02453	
Q9Y697	NFS1	0	53|74	2.7	0.13895	
Q5T6F2	UBAP2	0	41|52	5.9	0.04155	SG [47]
Q9Y5X1	SNX9	0	40|57	2.3	0.08151	
Q8TD19	HNEK9	0	38|38	3.4	0.03882	
Q9HCJ0	TNRC6C	0	38|17	1	0.01627	SG and PB [47]
Q13685	AAMP	0	37|43	1	0.09217	
Q9UKV8	EIF2C2 (Ago2)	0	35|42	5.5	0.04326	SG and PB [47]
Q9NVZ3	NECAP2	0	35|33	1	0.12928	
Q8NDV7	TNRC6A (GW182)	0	24|49	1.6	0.01860	SG and PB [47]
Q86UK7	ZNF598	0	24|20	2.6	0.02434	SG and PB [47]
Q96EK7	FAM120B	0	23|31	1.3	0.02967	
Q9UL18	EIF2C1 (Ago1)	0	23|23	2.6	0.02684	SG and PB [47]
Q8TF46	DIS3L	0	23|15	1	0.01803	SG and PB [47]
Q9Y5A6	ZSCAN21	0	18|15	1	0.03488	
Q14934	NFATC4	0	17|9	1	0.01441	
Q9HD34	LYRM4	0	14|18	1.5	0.17582	
P10253	GAA	0	11|10	1	0.01102	
Q96KG9	SCYL1	0	9|41	1	0.03094	
O75420	GIGYF1	0	10|19	1.8	0.01401	SG [47]
Q86V97	KBTBD6	0	7|11	1.5	0.01335	
Q9H8W3	FAM204A	0	9|10	1.3	0.04077	
Q9P2E3	ZNFX1	0	13|6	0	0.00495	SG [50]

*Average spectral counts from the two experiments divided by the protein length in amino acids, shown as a measure of protein recovery independent of length/peptide numbers.

^t^RISC components are marked in grey

**Fig 1:**
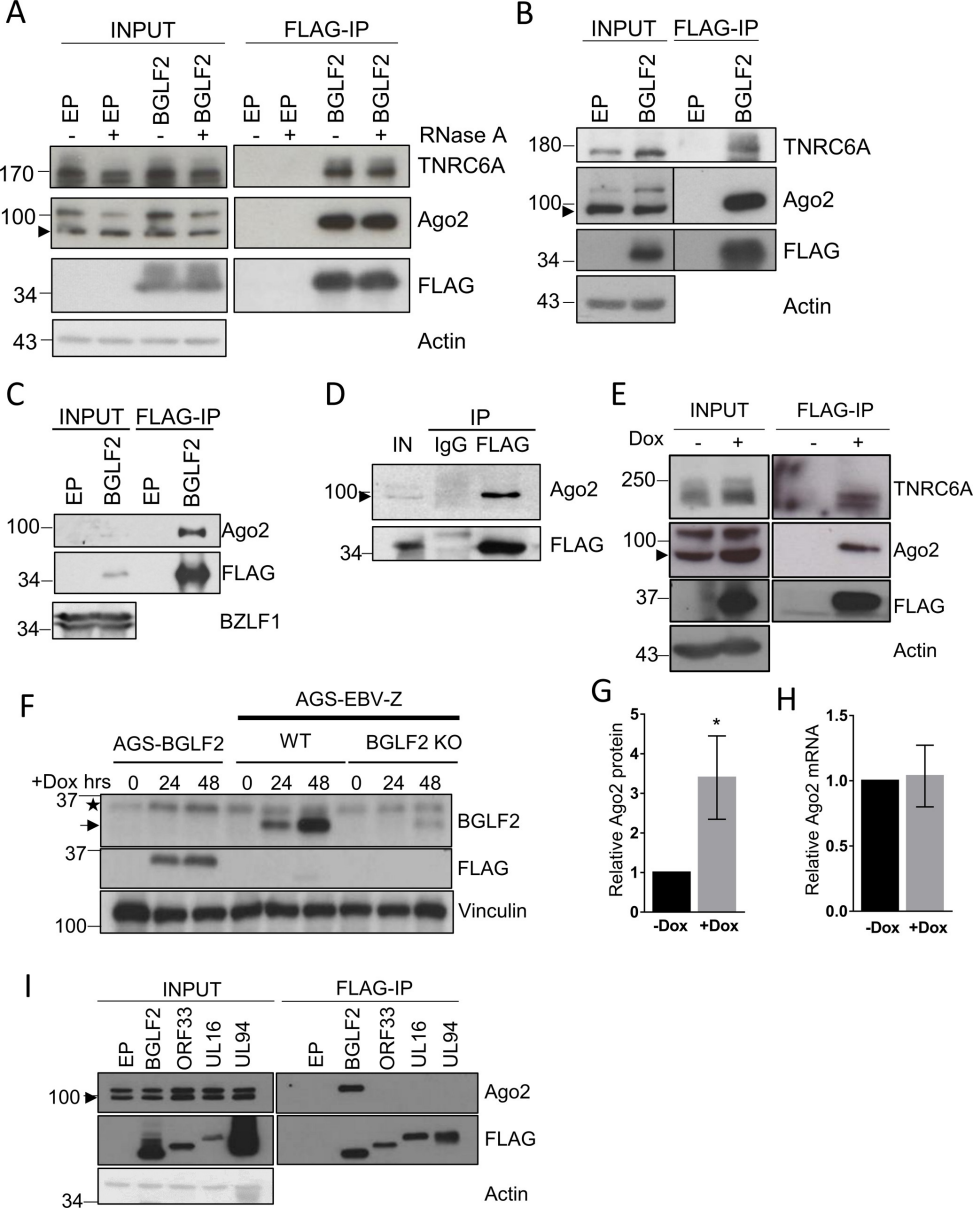
BGLF2 interacts with RISC. (A) 293T cells were transfected with pCMV3FC-BGLF2 (BGLF2) or pCMV3FC empty plasmid (EP) control and lysed 48 hours later. Cell lysates were treated with 20 μg/ml RNase A for 30 min or left untreated, as indicated, followed by IP using anti-FLAG resin. Western blotting was performed using antibodies against Ago2, TNRC6A, FLAG, or actin. 5% of the samples were run as inputs. Arrowhead indicates the Ago2 protein band. (B) AGS were transfected and processed as in A, except all samples include an RNase A treatment. 2.5% of the samples were run as inputs. (C) AGS-EBV-Z cells were transfected with pCMV3FC-BGLF2 or pCMV3FC then treated with Dox to induce EBV reactivation. Cells were lysed 24 hours later and FLAG IPs were performed followed by Western blotting for FLAG and Ago2. 2% of the lysates were run as inputs. A BZLF1 blot is also shown as proof of reactivation, where the upper band is the Tet-induced recombinant BZLF1 and the lower band is BZLF1 produced from the EBV. (D) Raji cells transfected with pCMV3FC-BGLF2 were treated with TPA and NaB to reactivate EBV, then lysed 24 hours later. The cell lysate was then divided in two and IPs performed with IgG- or anti-FLAG-conjugated beads. Western blotting was performed using antibodies against Ago2 and FLAG. 2% of the lysate was run as the input (IN) sample. (E) AGS-BGLF2 cells were treated with Dox for 48 hours to induce BGLF2-FLAG expression or left untreated, then processed as in B. (F) AGS-BGLF2 cells and AGS-EBV-Z cells with WT or BGLF2 KO EBV were treated with Dox for 0, 24 or 48 hours as indicated, then lysed in 9M urea and Western blotted using antibodies against BGLF2, FLAG and vinculin (loading control). The BGLF2 blot detects the FLAG-tagged BGLF2 in the AGS-BGLF2 cells (asterisk) as well as the endogenous BGLF2 produced from the EBV in AGS-EBV-Z cells (arrow). Note that there is a faint background band picked up by the BGLF2 antibody that is similar in size to FLAG-tagged BGLF2 and is seen in all the lanes. (G) AGS-BGLF2 cells were treated with Dox or left untreated as in B, then lysed in 9M urea and Western blotted for Ago2 and actin. Ago2 bands were quantified relative to actin and plotted relative to the uninduced (- Dox) sample. Average values and standard deviation are shown from three independent experiments, where * = 0.01 <P≤0.05. (H) Total RNA was extracted from AGS-BGLF2 cells treated with Dox for 48 hours or left untreated, and Ago2 transcripts were measured relative to actin transcripts and plotted as in D. (I) 293T cells were transfected with plasmids expressing FLAG-tagged BGLF2, KSHV ORF33, HSV-1 UL16, HCMV UL94, or pCMV3FC empty plasmid (EP). Forty-eight hours later, FLAG IPs were performed followed by Western blotting using antibodies against FLAG and Ago2. 2.5% of samples were run as inputs.

To validate the interaction with RISC, we expressed BGLF2-FLAG in 293T cells, then performed FLAG immunoprecipitations (IP) and Western blotting for endogenous Ago2 and TNRC6A. Since RISC interacts with RNA, we performed these IPs with and without RNase A treatment to determine if the interaction is RNA-mediated. As shown in Fig 1A, BGLF2 recovered Ago2 and TNRC6A, and these interactions were not affected by RNase A treatment, indicating that they were not RNA-mediated. We then performed similar BGLF2-FLAG IPs (with RNase A treatment) in AGS gastric carcinoma cells, commonly used to study EBV lytic infection, and again confirmed recovery of Ago2 and TNRC6A (Fig 1B). We also confirmed the interaction of BGLF2-FLAG with Ago2 in EBV lytic infection in AGS-EBV cells (Fig 1C), which contain a Tet-inducible BZLF1 lytic switch protein enabling induction of lytic infection in all of the cells by the addition of doxycycline (Dox; AGS-EBV-Z cells described in [22]). Similarly, BGLF2-FLAG was confirmed to interact with Ago2 in Raji Burkitt’s lymphoma cells, reactivated to the lytic cycle by sodium butyrate/TPA treatment (Fig 1D).

Transfection can result in high levels of protein expression due to the large number of plasmids taken up. Therefore, we also wanted to confirm the BGLF2-RISC interaction (and subsequent effects) with lower BGLF2 expression levels. To this end, we generated an AGS cell line containing a Tet-inducible BGLF2-FLAG (AGS-BGLF2) and showed that FLAG-IPs performed after BGLF2 induction by Dox treatment recovered Ago2 and TNRC6A (Fig 1E). We confirmed that the expression level of Dox-induced BGLF2-FLAG was physiological by comparing to BGLF2 levels in EBV infection. Dox-induced BGLF2 levels were found to be somewhat lower than that seen 24–48 hours after lytic reactivation of AGS-EBV-Z cells (Fig 1F), confirming that the BGLF2-FLAG is not overexpressed. In addition, we noticed that induction of BGLF2 expression resulted in increased Ago2 (and sometimes TNRC6A) protein levels (see Fig 1E, input samples), which might be a consequence of the BGLF2 interaction stabilizing this protein. To further explore this possibility, we quantified Ago2 proteins and Ago2 transcripts from multiple experiments, with and without induced expression of BGLF2-FLAG. We found that BGLF2 expression resulted in a consistent increase in Ago2 proteins without affecting Ago2 mRNA abundance (Fig 1G and 1H), supporting the hypothesis that BGLF2 affects Ago2 at the protein level (possibly due to stabilization by BGLF2 binding) and not the transcriptional level.

### RISC interaction is not conserved in BGLF2 homologues in other herpesviruses

All herpesviruses contain a homologue of BGLF2, which appear to have conserved functions in virion generation and infectivity [29,51–54]. Whether these proteins have similar roles in modulating cellular processes is unclear. We investigated whether BGLF2 homologues from three human herpesvirus, Kaposi’s sarcoma-associated herpesvirus (KSHV) ORF33, herpes simplex virus 1 (HSV-1) UL16, and human cytomegalovirus (HCMV) UL94, also interacted with RISC. To this end, FLAG-IPs were performed with FLAG-tagged KSHV ORF33, HSV-1 UL16, and HCMV UL94 expressed in 293T cells, followed by Western blotting for endogenous Ago2 (Fig 1I). Unlike BGLF2, none of these BGLF2 homologous were found to interact with Ago2, indicating that this interaction is a unique feature of EBV BGLF2.

### BGLF2 co-localizes with RISC in stress granules

**Fig 2:**
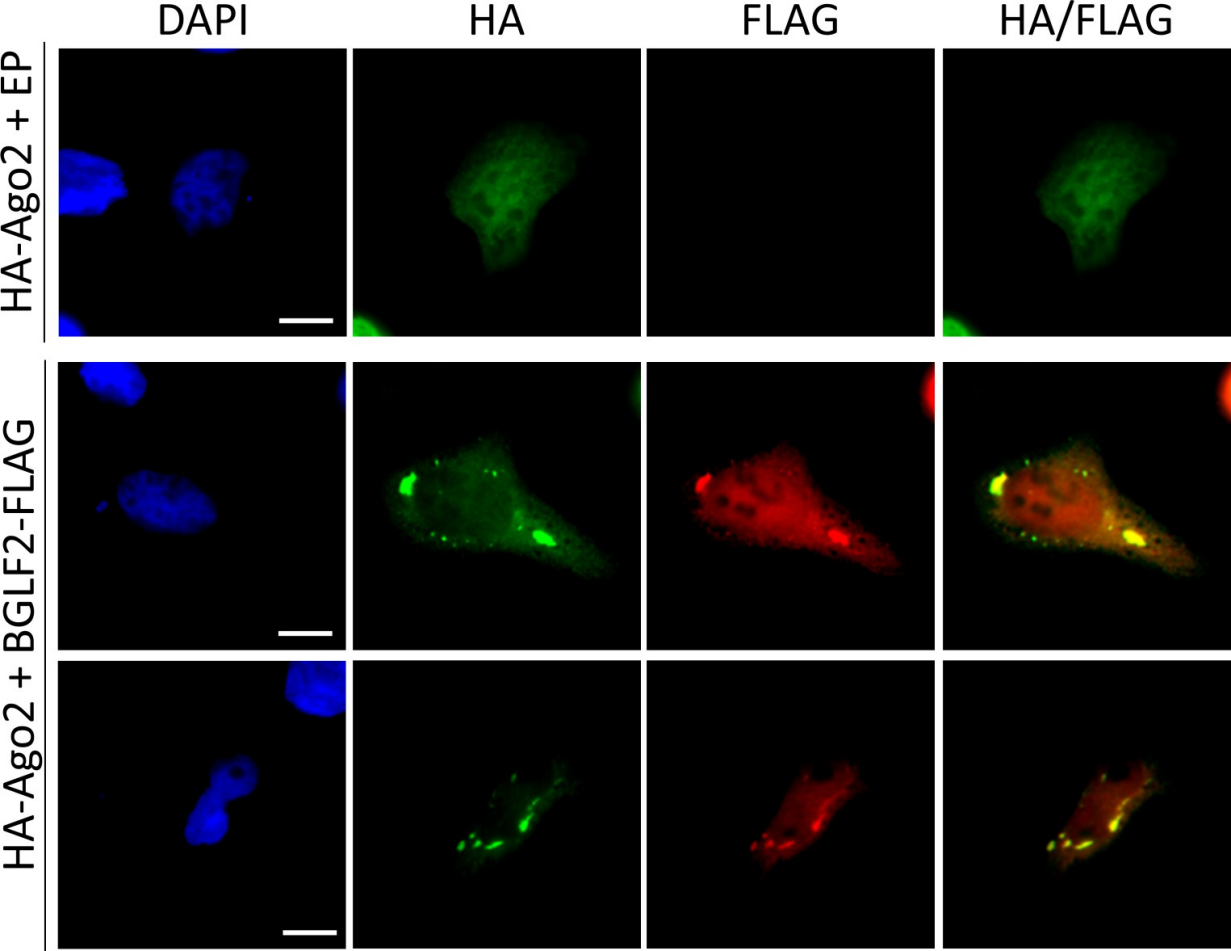
BGLF2 colocalizes with Ago2 foci. HONE-1 cells were co-transfected with a plasmid expressing HA-Ago2 and either pCMV3FC-BGLF2 or pCMV3FC empty plasmid control (EP). Twenty-four hours later, cells were fixed and stained with DAPI, anti-FLAG and anti-HA antibodies. Scale bar = 10 μm.

**Fig 3:**
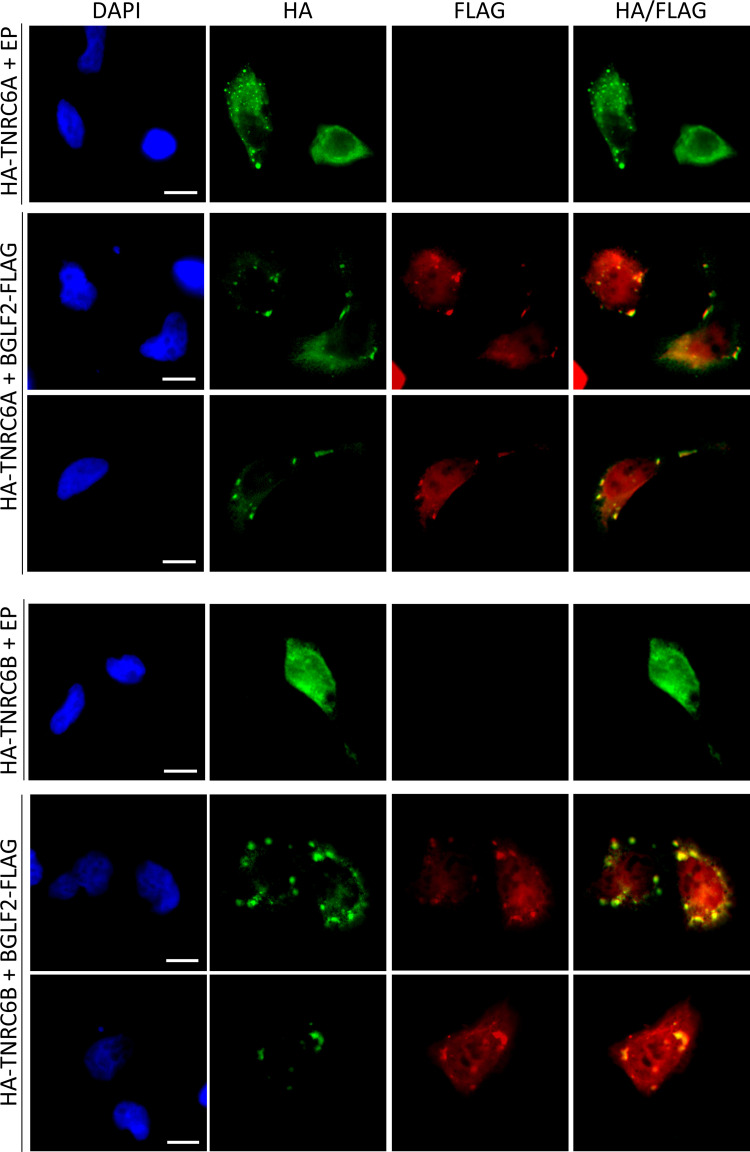
BGLF2 colocalizes with TNRC6 foci. HONE-1 cells were co-transfected with a plasmid expressing either HA-TNRC6A (upper panel) or HA-TNRC6B (lower panel) and either pCMV3FC-BGLF2 or pCMV3FC empty plasmid control (EP). Twenty-four hours later, cells were fixed and stained with DAPI, anti-FLAG and anti-HA antibodies. Scale bar = 10 μm.

**Fig 4:**
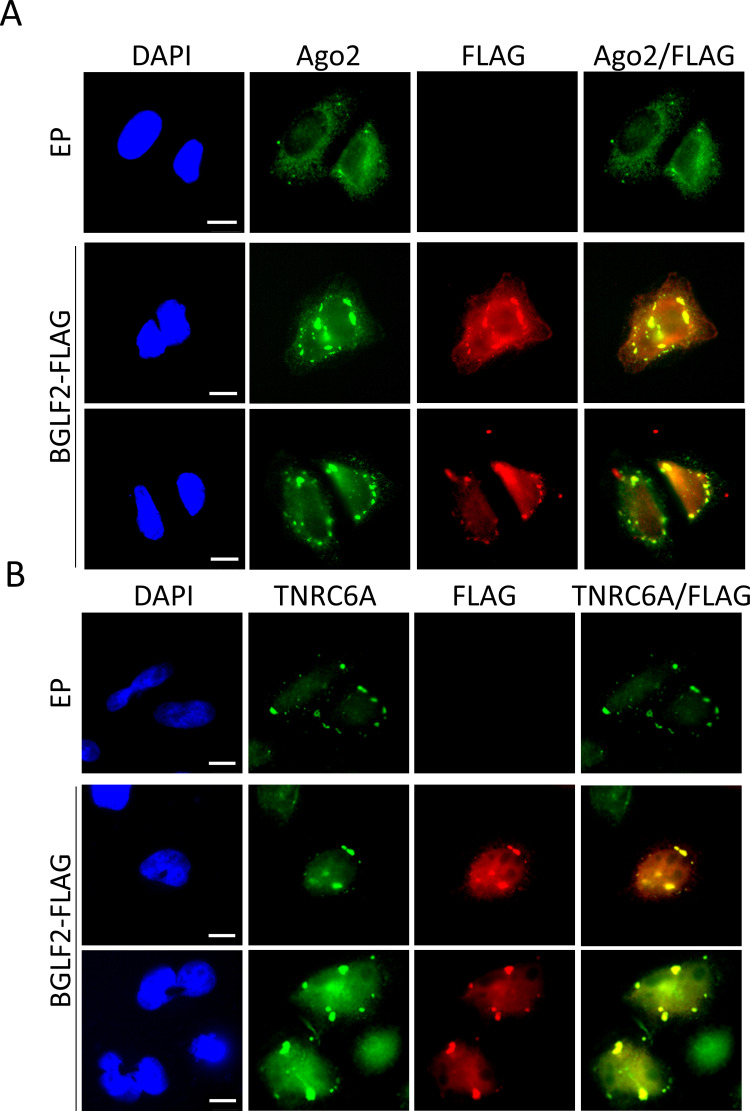
BGLF2 co-localizes with endogenous RISC components. HeLa cells were transfected pCMV3FC-BGLF2 or pCMV3FC empty plasmid control (EP) prior to being fixed and stained with DAPI and anti-FLAG antibodies and antibodies against either Ago2 (A) or TNRC6A (B). Scale bar = 10 μm.

In addition to IP experiments, we also examined whether BGLF2 localized with RISC in cells, using two approaches. First, we expressed HA-tagged RISC components (Ago2, TNRC6A TNRC6B) with or without BGLF2-FLAG in HONE-1 (EBV-negative nasopharyngeal carcinoma) cells and stained the cells for HA and FLAG (Figs 2 and 3). Each of the RISC components generated some cytoplasmic foci in addition to more diffuse staining. When BGLF2 was co-expressed with them, it generated similar patterns, with some of the BGLF2 co-localizing with RISC subunits in cytoplasmic foci. Secondly, we examined co-localization of BGLF2-FLAG with endogenous RISC proteins. This was performed in HeLa cells, since they are most commonly used to visualize RISC-associated bodies [55,56]. When HeLa cells expressing BGLF2-FLAG were stained with antibodies against FLAG and Ago2 (Fig 4A) or TNRC6A (Fig 4B), BGLF2 was again found to co-localize with Ago2 and TNRC6A in cytoplasmic bodies. The localization of endogenous Ago2 to large bodies (typical of those formed by BGLF2) was not seen in the absence of BGLF2.

**Fig 5:**
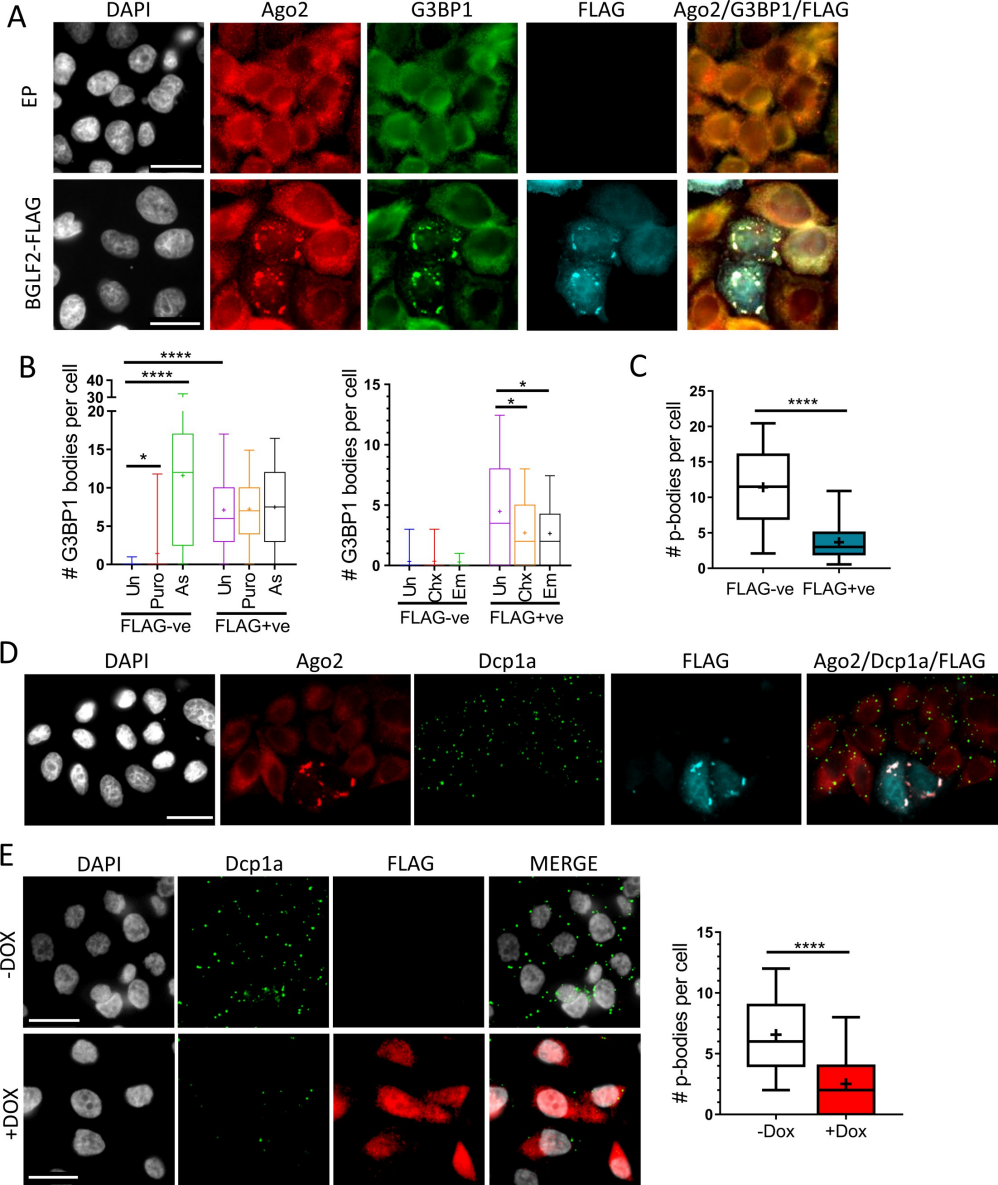
BGLF2 co-localizes with Ago2 in stress granules and induces loss of p-bodies. (A) HeLa cells were transfected pCMV3FC-BGLF2 or pCMV3FC empty plasmid control (EP) prior to being fixed and stained with DAPI and antibodies against FLAG, Ago2 and G3BP1 (stress granule marker) (B) HeLa cells were transfected and processed as in A, except prior to fixing, cells were either left untreated (Un), or treated with puromycin (Puro), sodium arsenite (As), cycloheximide (Chx), or emetine (Em). The number of G3BP1 bodies per cell was counted in 50 FLAG negative (FLAG-ve) and 50 FLAG positive (FLAG+ve) cells per treatment. Median values are shown by the horizontal line and average values are indicated by the +. Boxes mark the 25th to 75th percentile, while whiskers indicate 5th to 95th percentile. * = 0.01<P≤0.05, **** = P≤0.0001. (C) HeLa cells were transfected and processed as in A, except that cells were stained for Dcp1a (p-body marker) instead of G3BP1. The number of p-bodies per cell was counted for 50 FLAG negative (FLAG-ve) and 50 FLAG positive (FLAG+ve) cells. (D) Representative image from C showing reduction in Dcp1a foci in FLAG positive cells relative to FLAG negative cells. (E) AGS-BGLF2 cells were treated with Dox for 24 hours (+Dox) or left untreated (-Dox) prior to being fixed and stained with DAPI and antibodies against FLAG and Dcp1a. The number of p-bodies per cell for 100 cells with and without Dox were counted. Scale bars = 25 μm.

RISC is known to be associated with two types of cytoplasmic membraneless organelles, stress granules and p-bodies, which can be identified by staining with antibodies against G3BP1 or Dcp1a, respectively. To determine if the BGLF2 foci correspond to stress granules, we repeated the immunofluorescence microscopy for BGLF2-FLAG and endogenous Ago2 but also stained for G3BP1. In the absence of BGLF2-FLAG, G3BP1 has a diffuse cytoplasmic localization reflecting the lack of stress granules (Fig 5A). This is expected as stress granules normally require induction with a stressor, such as sodium arsenite or puromycin, and control treatments with these chemicals confirmed that stress granules could be induced. (Fig 5B). However, in cells expressing BGLF2, G3BP1 foci were observed that co-localized with Ago2 and BGLF2, in the absence of any chemical treatment (Fig 5A). Therefore, the BGLF2 foci that contain RISC have characteristics of stress granules. This was consistent with the AP-MS data that identified several proteins which localize to stress granules. Quantification of the number of stress granules in cells expressing or not expressing BGLF2 showed that BGLF2 induced stress granules in these cells, at a level in between that induced with puromycin and sodium arsenite (Fig 5B). In addition, the number of BGLF2-induced bodies decreased by cycloheximide and emetine treatments, as expected of stress granules. However, BGLF2 only appears to form these bodies in cells that are able to form stress granules. For example, we have not seen any stress granule induction in AGS cells in response to sodium arsenite (which kills these cells) or puromycin (S1A Fig) treatments, nor does BGLF2 form cytoplasmic bodies in these cells (Fig 5E).

### BGLF2 induces the loss of p-bodies

We also investigated the association of BGLF2 with p-bodies, which are present in all cells, using Dcp1a as a marker. Although we did not see any obvious enrichment of BGLF2 at p-bodies in HeLa cells, we noticed that the number of these bodies was reduced in the presence of BGLF2 (Fig 5C and 5D). This effect was verified by counting the number of p-bodies per cell in cells expressing or not expressing BGLF2, showing a 4-fold reduction in p-bodies in the presence of BGLF2. A similar effect on p-bodies was seen when BGLF2 was expressed in HONE-1 cells (S1B Fig). We also investigated this effect in the AGS cells with Tet-inducible BGLF2, which have low levels of BGLF2 expression. Dox-induction of BGLF2 for 24 hours led to 3-fold reduction in p-bodies (Fig 5E). We conclude that BGLF2 consistently induces the loss of p-bodies, which would be expected to disrupt miRNA function, and that formation of stress granules is not a requirement for this p-body disruption.

### BGLF2 interferes with let-7 miRNA function

**Fig 6:**
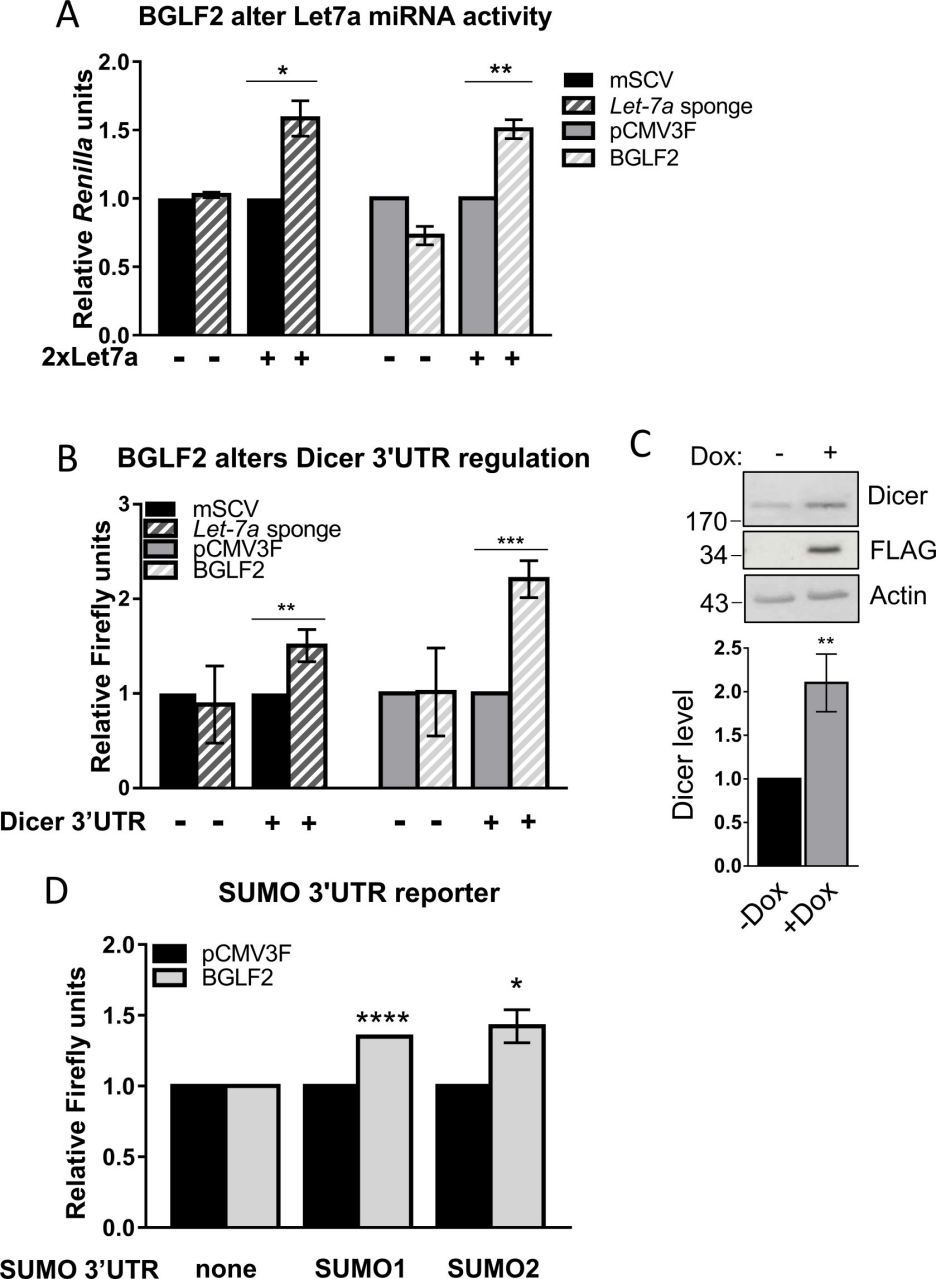
BGLF2 inhibits let-7a function in reporter assays. (A) HONE-1 were co-transfected with a *Renilla* luciferase reporter with or without let-7 binding sites (+ or– 2xLet7a), a firefly luciferase control plasmid and either mSCV-based let-7 sponge plasmids, mSCV empty plasmid, pCMV3FC-BGLF2 or pCMV3FC empty plasmid as indicated. *Renilla* and firefly luciferase were quantified and *Renilla* values were normalized to firefly values. (B) The same experiment as in A except that a firefly luciferase reporter containing the Dicer 3’UTR (+ Dicer 3’UTR) or no UTR (- Dicer 3’UTR) was used and compared to a *Renilla* luciferase negative control plasmid. (C) AGS-BGLF2 cells were treated with Dox (+) for 48 hours to induce BGLF2-FLAG expression or left untreated (-), then lysed in 9M urea and Western blotted for Dicer, FLAG and actin (Top). Dicer bands were quantified relative to actin and induced samples (+Dox) were plotted relative to the uninduced (-Dox) sample (Bottom). Average values and standard deviation are shown from three independent experiments. (D) HONE-1 cells were co-transfected with a firefly luciferase reporter containing SUMO1 3’UTR, SUMO2 3’UTR or no 3’UTR (pGL4.10; as indicted), a *Renilla* luciferase control plasmid, and either pCMV3FC-BGLF2 or pCMV3FC (empty plasmid). Firefly luciferase activity was normalized to *Renilla* luciferase activity for each sample. For each luciferase assay, average values from 3 independent experiments (with standard deviation) are shown relative to the value for the empty plasmid. * = 0.01<P≤0.05, ** = 0.001<P≤0.01, *** = 0.0001<P≤0.001, **** = P≤0.0001.

The ability of BGLF2 to interact with RISC and disrupt p-bodies suggests that BGLF2 might affect the ability of RISC to mediate miRNA function. We tested this hypothesis by examining effects of BGLF2 on the well-studied let-7 family of miRNAs. To this end, we used a *Renilla* luciferase reporter containing two tandem let-7a binding sites (2xLet7a). We first confirmed that this reporter was regulated by let-7 miRNAs by transfecting it with and without a let-7 sponge construct that ursurps let-7 miRNAs, thereby reducing targeting of the luciferase reporter [57]. As expected, this sponge resulted in an increase in the *Renilla* luciferase activity (Fig 6A). The *Renilla* luciferase activity of the 2xLet7a reporter was then compared with and without BGLF2 expression and normalized to effects on a firefly luciferase construct lacking let-7a sites (an internal control). BGLF2 consistently increased *Renilla* luciferase expression to a similar extent as the let-7 sponge (Fig 6A) and dependent on the presence of the let-7 sites. This suggested that BGLF2 interferes with let-7a miRNA function.

We further examined the effect of BGLF2 on let-7 miRNAs by using a firefly luciferase reporter with and without a 3’UTR from the Dicer gene, which contains multiple let-7 miRNA binding sites and has been shown to be regulated by let-7 miRNAs [58,59]. We confirmed regulation of this reporter by let-7 miRNAs using the let-7 sponge and then tested the effect of co-expression with BGLF2 (using a *Renilla* luciferase reporter lacking the Dicer 3’UTR as an internal control; Fig 6B). Consistent with the 2xLet7a reporter results, BGLF2 significantly increased firefly luciferase levels in the reporter containing the Dicer 3’UTR, but not in reporters lacking this sequence, providing further evidence that BGLF2 disrupts let-7 miRNAs activity. To determine whether this up-regulation of Dicer by BGLF2 also occurred in endogenous Dicer, we compared Dicer protein levels in AGS cells with Tet-inducible BGLF2, before and after BGLF2 expression. Consistent with the reporter results, Dicer levels increased after BGLF2 expression (Fig 6C).

### BGLF2 increases SUMO proteins through miRNA effects

**Fig 7:**
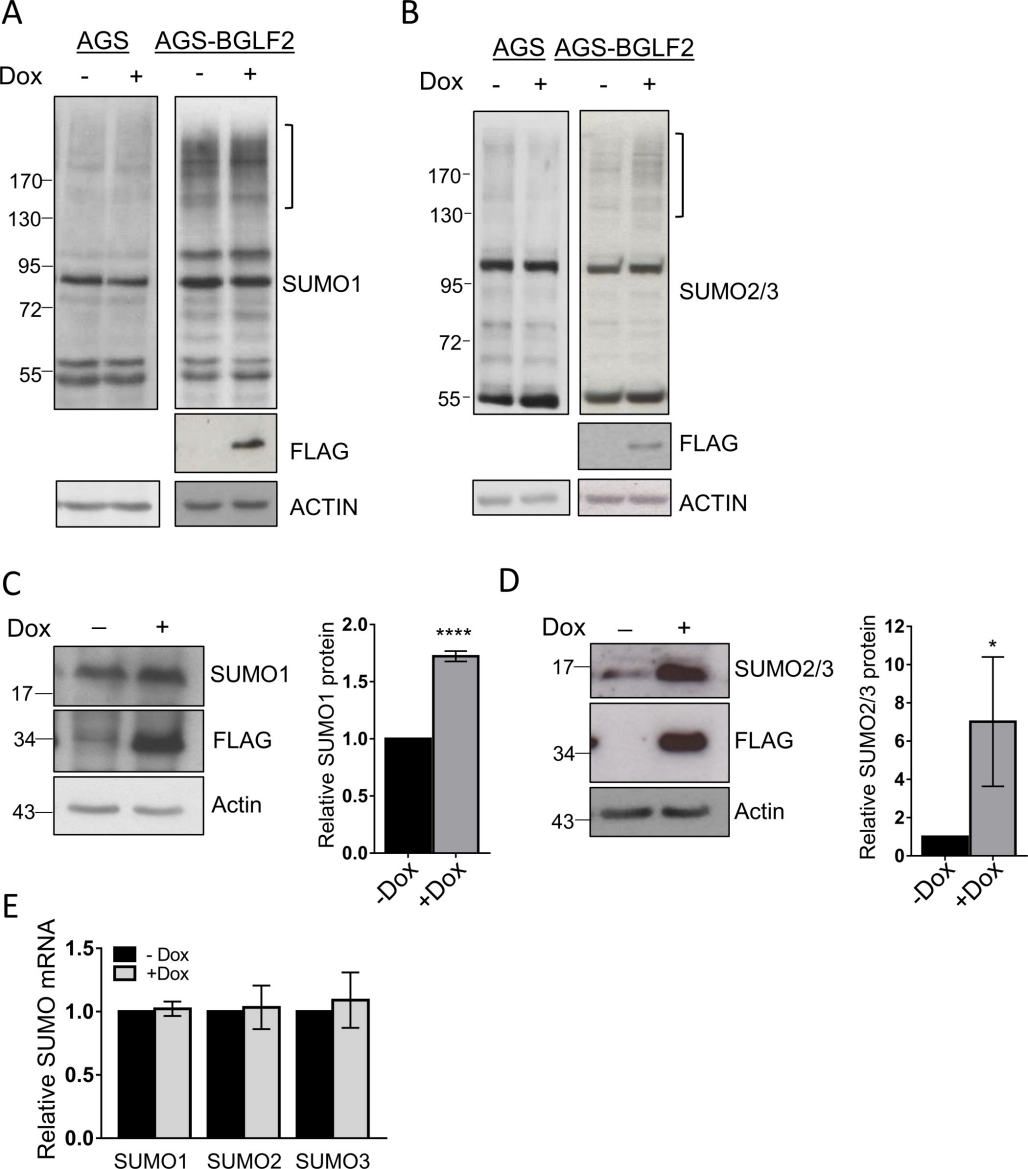
BGLF2 induces SUMOylation and increases free SUMO post-transcriptionally. (A) and (B) AGS or AGS-BGLF2 cells were treated with Dox for 48 hours or left untreated. Whole cell lysates were then analysed by Western blotting using antibodies against FLAG, actin and either SUMO1 (A) or SUMO2/3 (B). The bracket marks the position of cellular proteins with increased SUMOylation with BGLF2. (C) and (D) Lysates from A and B were analysed for free SUMO by Western blotting using a higher percentage gel and antibodies against FLAG, actin and either SUMO1 (C) or SUMO2/3 (D) Free SUMO bands were quantified from 3 independent experiments and average values for the induced (+Dox) samples were plotted relative to the uninduced (-Dox) samples which were set to one. * = 0.01<P≤0.05, **** = P≤0.0001. (E) Samples generated as in A and B were analysed for SUMO1, SUMO2, or SUMO3 transcripts by qRT-PCR. Transcripts were normalized to actin transcript levels and average values for induced (+Dox) samples from 3 independent experiments are shown relative to uninduced (-Dox) samples which were set to one.

BGLF2 was previously shown to globally increase SUMOylation of cellular proteins in multiple cell lines [23]. One mechanism by which SUMOylation can be globally increased is by increasing the level of free SUMO [60,61]. Interestingly, SUMO1, SUMO2, and SUMO3 transcripts all contain multiple let-7 recognition sites and have been shown to be regulated by let-7 miRNAs [61]. Since BGLF2 can interfere with let-7 function, we investigated the possibility that this interference results in increased free SUMO levels which may drive SUMOylation. We first confirmed that BGLF2 induced SUMOylation in the Tet-inducible AGS-BGLF2 cells by preparing whole cell lysates with and without Dox treatment and Western blotting using antibodies against SUMO1 (Fig 7A) or SUMO2/3 (Fig 7B). Consistent with previous studies [23], BGLF2 expression led to an increase in SUMOylated cellular proteins, which was more obvious for SUMO2/3 than for SUMO1. As a control to confirm the requirement of BGLF2, parental AGS cells lacking the BGLF2 cassette were also treated with Dox and processed in the same manner, but as expected, this treatment did not lead to increased SUMOylation. The lysates from the AGS-BGLF2 cells were then reanalysed (on a higher percentage gel) for free SUMO. BGLF2 was found to consistently increase the levels of free SUMO1 (Fig 7C) and SUMO2/3 (Fig 7D), with a larger effect on SUMO2/3. This mirrors the effect on SUMOylation and suggests that the increase in SUMOylation caused by BGLF2 may be due to increased SUMO protein levels.

We then investigated whether the increase in free SUMO could be due to BGLF2 effects on miRNA. We first examined whether BGLF2 expression affected the level of SUMO transcripts by collecting total RNA from the same cells used for Western blotting and performing RT-qPCR for SUMO1, SUMO2, SUMO3, and actin transcripts, the later of which was used as a normalization control. Unlike the effect of BGLF2 on SUMO proteins, SUMO transcripts were unaffected by BGLF2 (Fig 7E), indicating that the regulation of SUMO levels occurred post-transcriptionally.

We next investigated whether BGLF2 regulates SUMO transcripts through their 3’UTRs, which are known to be regulated by multiple let-7 miRNAs [61]. To this end, we generated firefly luciferase reporters containing fragments of the SUMO1 or SUMO2 3’UTRs and examined effects of co-expression with BGLF2, using a *Renilla* luciferase reporter lacking 3’-UTRs as a normalization control. BGLF2 was found to consistently increase firefly luciferase reporter activity (Fig 6D), suggesting that BGLF2 regulates SUMO transcripts through their 3’UTRs. Since these sequences contain let-7 recognition motifs, the results are consistent with BGLF2 increasing SUMO expression by interfering with let-7 miRNAs.

### BGLF2 inhibits let-7 interaction with Ago2

**Fig 8:**
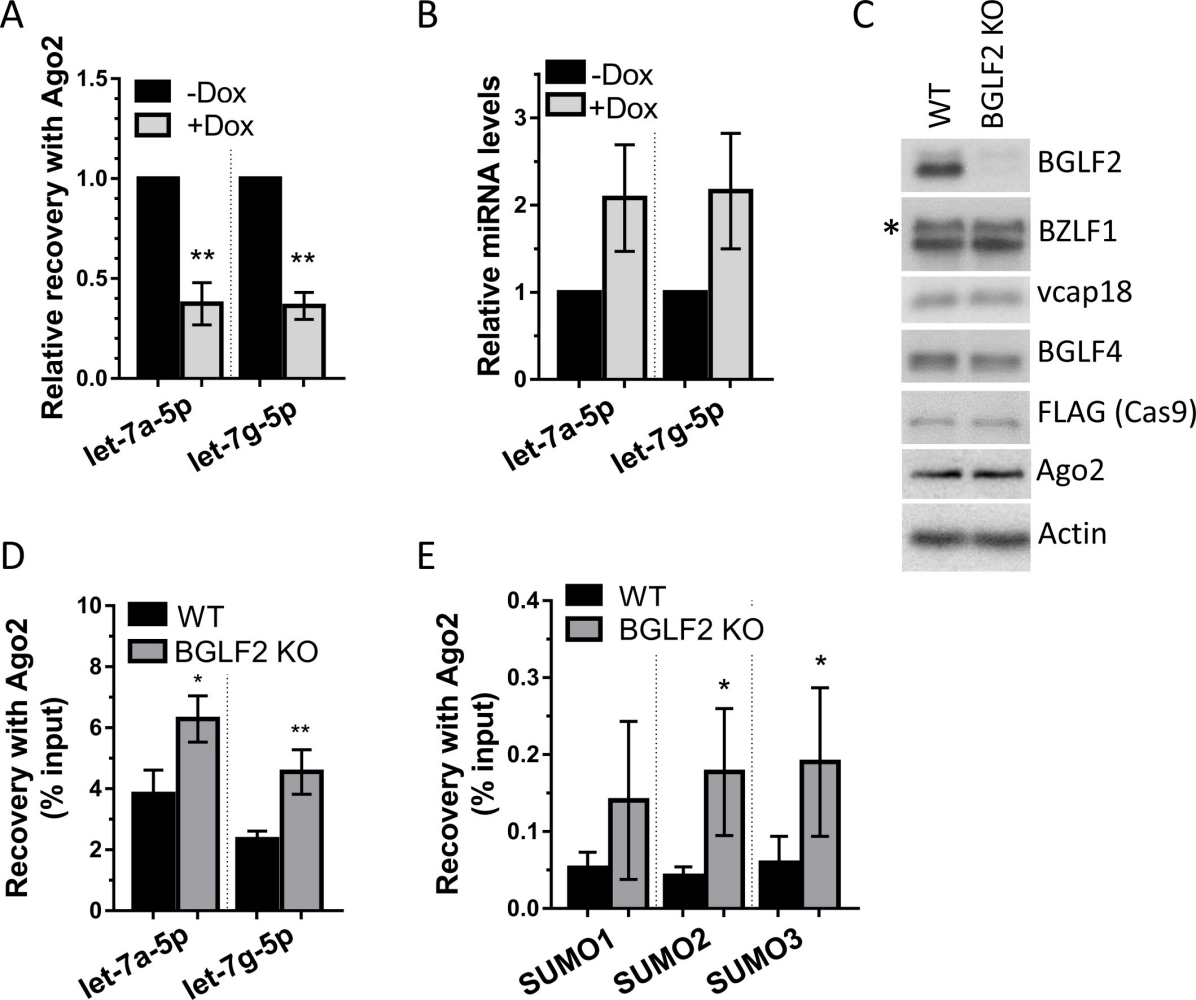
BGLF2 inhibits let-7 miRNA incorporation into Ago2. (A) Ago2 was immunoprecipitated from AGS-BGLF2 cells treated (+Dox) or untreated (-Dox) with Dox using anti-Ago2 antibody or IgG negative control antibody. Recovered let-7a-5p and let-7g-5p miRNAs were quantified by RT-qPCR, normalized to spike in UniSp6 synthetic RNA control and calculated as fold enrichment in Ago2-IP over IgG control IP. Values were also normalized to recovery of Ago2 protein in the IP. Average values for +Dox samples from 3 independent experiments are shown relative to -Dox samples, which were set to 1. (B) let-7a-5p and let-7g-5p miRNAs from cell lysates in A (prior to immunoprecipitations) were quantified by RT-qPCR, normalized to U6 RNA and plotted as in A. (C) AGS-EBV-Z cell pools containing a non-targeting (WT) or BGLF2-targeting (BGLF2 KO) CRISPR/Cas9 were reactivated by Dox treatment for 48 hours followed by Western blotting using antibodies against the EBV proteins BGLF2, BZLF1, vcap18, and BGLF4, as well as FLAG (to detect FLAG-tagged Cas9), Ago2, and actin. Two BZLF1 bands are detected from the endogenous virus and Tet-inducible expression cassette (asterisk). (D) Ago2 immunoprecipitations were performed as in A using lysates generated as in C. Let-7a-5p and let-7g-5p miRNAs were quantified in lysates (input) and Ago2 IP samples and normalized to spike in UniSp6 synthetic RNA. Let-7a-5p and let-7g-5p values in Ago2-IP samples were also normalized to Ago2 protein recovered in each IP. Average values for 3 independent experiments are shown as percentage of input let-7a-5p and let-7g-5p miRNA. (E) Ago2 immunoprecipitations were performed as in D. and transcripts for SUMO1, SUMO2 and SUMO3 were quantified by RT-qPCR and normalized to Ago2 protein abundance in each IP. Average values for 4 independent experiments are shown as percentage of SUMO transcripts in the input. * = 0.01<P≤0.05, ** = 0.001<P≤0.01.

We next further investigated the mechanism by which BGLF2 inhibits let-7 function. Our model was that BGLF2 sequesters RISC, making it less available to interact with cellular miRNAs. We tested this model by determining how BGLF2 expression affects the association of let-7 miRNAs with Ago2. To this end, Ago2-IPs were performed in AGS-BGLF2 cells with and without induction of BGLF2 (compared to an IgG control) and recovered miRNAs were quantified by RT-qPCR, using primers specific to let-7a-5p and let-7g-5p miRNAs, both of which regulate SUMO1, SUMO2 and SUMO3 3’UTRs [61]. We then compared the amount of let-7a-5p and let-7g-5p recovered with Ago2 as compared to the IgG control, in the presence and absence of BGLF2. These values were also normalized to Ago2 recovery, as determined by Western blotting of the same samples (S2A Fig). As shown in Fig 8A, the degree of recovery of both let-7 miRNAs with Ago2 decreased about 3-fold in the presence of BGLF2. We also checked the total level of these let-7 miRNAs in the lysates prior to Ago2-IP, to determine if the decreased recovery with Ago2 could be due to a decrease in the cellular levels of these miRNAs. However, the cellular levels of let-7a-5p and let-7g-5p were not decreased by BGLF2 but were somewhat increased (although not statistically significant; Fig 8B). The results support a model in which BGLF2 inhibits let-7 miRNA incorporation into Ago2.

We further investigated this model in the context of EBV lytic infection by comparing WT EBV to EBV with a BGLF2 knockout (KO). The BGLF2 KO was generated by lentivirus-delivered CRISPR/Cas9 targeting in the context of AGS-EBV-Z cells, which are AGS-EBV cells with a Tet-inducible BZLF1 cassette that enables efficient reactivation to the lytic cycle by Dox treatment [22]. These cells were compared to AGS-EBV-Z cells transduced with a negative control lentivirus expressing CRISPR/Cas9 but lacking a guide RNA. In both cases cell pools were used, and not individual clones, to ensure that a particular integration site of the lentivirus was not responsible for the effect. Reactivation of WT and BGLF2 KO virus with Dox confirmed that the KO virus produced little BGLF2 (Figs 1F and 8C), while other viral lytic proteins remained unaffected (Fig 8C). Comparison of the lysates also showed that Ago2 levels were unaffected by the BGLF2 KO. Ago2-IPs were performed with these lysates and recovered amounts of let-7a-5p and let-7g-5p miRNAs were determined as above and expressed as a percentage of the let-7a-5p and let-7g-5p miRNAs in the lysates. As shown in Fig 8D, the BGLF2 KO resulted in a consistent increase in let-7a-5p and let-7g-5p associated with Ago2. We further investigated this effect by asking if this increase in let-7 miRNAs incorporated into Ago2 resulted in the expected increase in associated SUMO transcripts. Although there was a lot of variability in recovery of SUMO transcripts, the recovery of SUMO2 and SUMO3 transcripts with Ago2 was significantly increased ~3-fold in the BGLF2 KO lysates (as compared to WT EBV; Fig 8E). This is consistent with the greater effect of BGLF2 on SUMO2/3 than SUMO1. These results further support a model in which BGLF2 inhibits let-7 miRNA function by interfering with the incorporation of these miRNAs into Ago2 complexes.

### Inhibition of Ago2-miRNA interactions by BGLF2 is not limited to let-7

**Fig 9:**
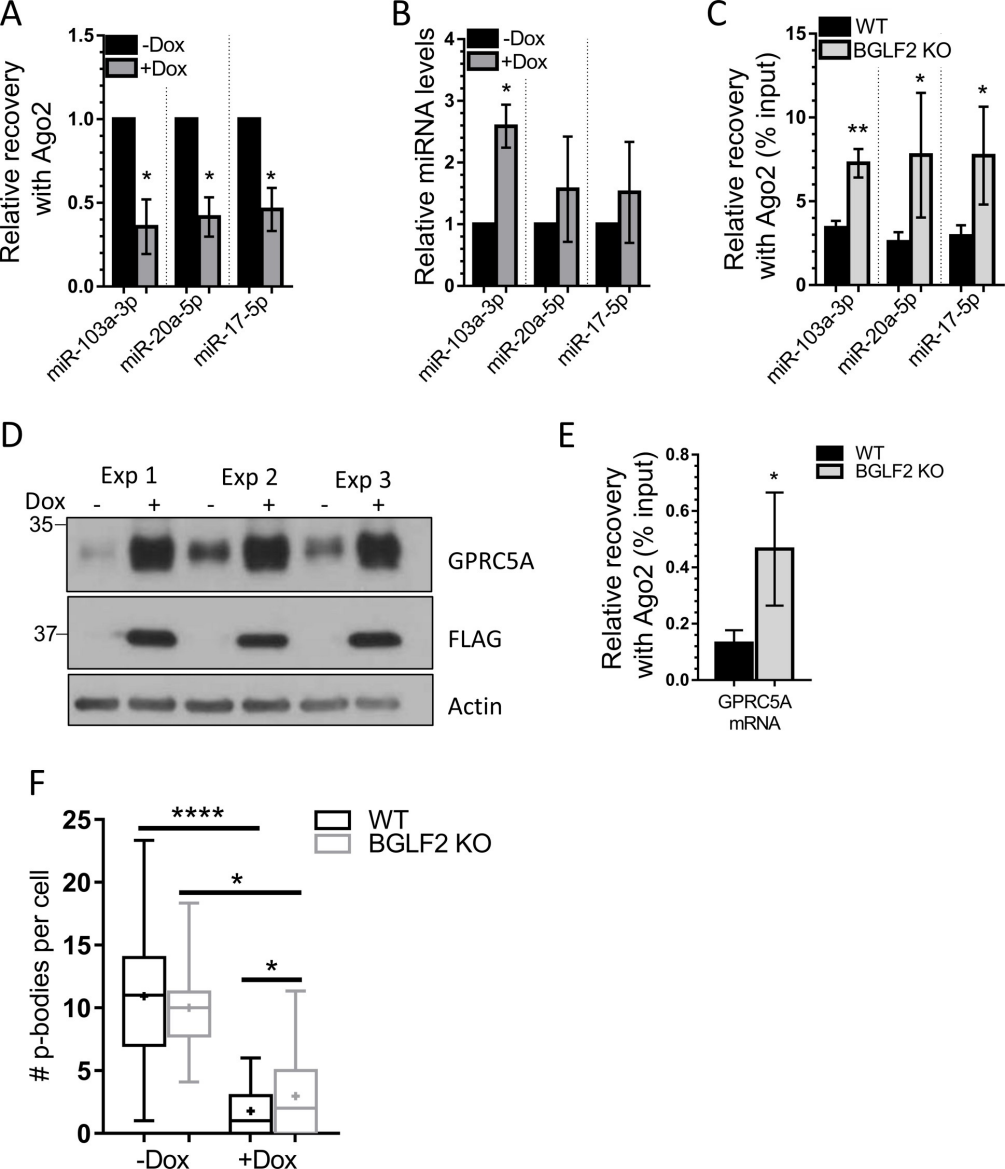
BGLF2 inhibits multiple miRNAs. (A) Ago2 was immunoprecipitated from lysates of AGS-BGLF2 cells treated (+Dox) or untreated (-Dox) with Dox using anti-Ago2 antibody or IgG negative control antibody. Recovered miR-103a-3p, miR-20a-5p, and miR-17-5p miRNAs were quantified by RT-qPCR, normalized to spike in UniSp6 synthetic RNA control and calculated as fold enrichment in Ago2-IP over IgG control IP. Values were also normalized to recovery of Ago2 protein in the IP. Average values for +Dox samples from 3 independent experiments are shown relative to -Dox samples, which were set to 1. (B) miR-103a-3p, miR-20a-5p, miR-17-5p miRNAs from cell lysates in A (used for immunoprecipitations) were quantified by RT-qPCR, normalized to U6 RNA and plotted as in A. (C) Ago2-IP were performed using AGS-EBV-Z cells as in Fig 8D. miR-103a-3p, miR-20a-5p, and miR-17-5p miRNAs were quantified in input lysates and Ago2 IP samples and normalized to spike in UniSp6 synthetic RNA. miR-103a-3p, miR-20a-5p, and miR-17-5p values in Ago2-IP samples were also normalized to Ago2 protein recovered in each IP. Average values for 3 independent experiments are shown as percentage of input miR-103a-3p, miR-20a-5p, and miR-17-5p miRNA. (D) AGS-BGLF2 cells were treated with Dox for 48 hours or left untreated, then lysed in 9M urea and Western blotted for GPRC5A, FLAG, and actin. (E) Ago2 immunoprecipitations were performed as in C and transcripts for GPRC5A were quantified by RT-qPCR and normalized to Ago2 protein recovery. Average values for 4 independent experiments are shown as percentage of GPRC5A transcripts in the input. (F) The number of p-bodies per cells in AGS-EBV-Z cell pools containing a non-targeting (WT) or BGLF2-targeted (BGLF2 KO) CRISPR/Cas9, which were reactivated with Dox (+Dox) or left untreated (-Dox). * = 0.01<P≤0.05, ** = 0.001<P≤0.01, **** = P≤0.0001.

The ability of BGLF2 to bind RISC and disrupt p-bodies suggests that BGLF2 may globally inhibit miRNA function. To investigate whether BGLF2 impacts miRNA other than those from the let-7 family, we repeated the Ago2 IP experiments in AGS cells with Tet-inducible BGLF2 and quantified recovery of three additional miRNAs; miR103a-3p, miR20a-5p and miR17-5p. All were found to have significantly decreased interactions with Ago2 in the presence of BGLF2, (Fig 9A) even though the cellular levels of these miRNAs were all somewhat increased with BGLF2 (Fig 9B). We also examined how the association of these miRNAs with Ago2 was affected by BGLF2 in the context of EBV infection. Comparison of Ago2 association in lytic infection with WT or BGLF2 KO EBV, showed that the interactions of all three miRNAs with Ago2 increased in the absence of BGLF2 (Fig 9C). miR103a-3p is known to target transcripts for GPRC5A [62]. Therefore, we examined how induction of BGLF2 expression in the AGS cells affected GPRC5A protein levels. Consistent with the affect on miR103a-3p, BGLF2 expression resulted in a major increase in GPRC5A expression (Fig 9D). In addition, in the context of EBV lytic infection, the association of GPRC5A transcripts with Ago2 was increased with the BGLF2 KO relative to WT EBV (Fig 9E). Finally, we also examined how p-bodies were affected in lytic EBV infection. Imaging of Dcp1a foci showed that lytic reactivation of WT EBV resulted in a large (6-fold) decrease in the number of p-bodies, and that, relative to WT infection, the p-body numbers increased 2-fold in lytic infection with the BGLF2 KO (Fig 9F). The results suggest that BGLF2 has global effects on miRNA function and that components of EBV infection in addition to BGLF2 disrupt p-bodies.

## Discussion

We have shown that the EBV tegument protein, BGLF2, interacts with cellular RISC and interferes with its function in enabling miRNA activity. BGLF2 has been previously shown to modulate several cellular processes, including upregulation of AP-1 promoters, downregulation of IFN and NF-κB signaling, cell cycle modulation and induction of SUMOylation [23,27,31–34]. Our finding raises the possibility that these and other cellular events may be modulated by BGLF2 through its effect on miRNAs. As an example, we have shown that BGLF2 interferes with the activity of let-7 miRNAs by inhibiting the loading of let-7 miRNAs into Ago2 proteins. Let-7 miRNA are known to target the 3’UTRs of SUMO transcripts and, in agreement with this, we showed that BGLF2 upregulates the level of free SUMO proteins, without affecting the levels of their transcripts, and acted on reporter constructs regulated by SUMO 3’UTRs or other UTRs with let-7 target sites. The resulting increase in free SUMO is a mechanism known to drive protein SUMOylation [60,61,63,64] and explains the induction of global SUMOylation by BGLF2.

We have shown that BGLF2 inhibits the function of let-7 miRNAs, and that one outcome is the upregulation of free SUMO driving increased SUMOylation. SUMOylation controls many processes, including oncogenesis and antiviral responses [65,66], raising the possibility that BGLF2 can manipulate these processes. Three other EBV proteins have also been shown to increase SUMOylation of cellular proteins, specifically the lytic proteins SM and BMRF1 [23] and latent membrane protein 1 (LMP1) [60,67–69]. SM has SUMO E3 ligase activity, while the mechanism of SUMOylation induction of BMRF1 is unknown. Like BGLF2, LMP1 appears to drive SUMOylation, at least in part, by increasing free SUMO protein levels, although LMP1 increases free SUMO at the transcriptional level [60].

Let-7 miRNAs target cellular mRNAs in addition to SUMO and are considered to be tumour suppressors due to their roles in regulating cell proliferation, survival and metabolism [70]. In addition, several roles for the let-7 miRNA family have been identified in innate and adaptive immunity [71]. Therefore, it is likely that BGLF2 would also impact these processes through its interference with let-7 activity. In addition, a role for let-7a has been identified in inhibiting EBV reactivation and promoting latency, so its downregulation by BGLF2 might promote lytic infection [72]. Let-7 miRNAs have also been found to be affected by the EBV latency protein EBNA1, however in that case, EBNA1 increases let-7a miRNA by transactivating the expression of let-7a primary RNAs. Therefore, it may be advantageous for EBV to upregulate let-7 miRNAs in latent infection and downregulate them in lytic infection.

BGLF2 is a virion tegument protein that is conserved in all three herpesviruses families and appears to have a conserved role in virion generation through final envelopment of the capsids [51,73–76]. However, the ability to commandeer RISC may be unique to BGLF2, since we were unable to detect any interactions of the KSHV (ORF33), HSV-1 (UL16) and CMV (UL94) homologues with Ago2. This is not surprising, given that these homologues only have 30.8% (ORF33), 13.7% (UL16) and 14.3% (UL94) identity with BGLF2 [73]. However, like BGLF2, another KSHV protein, ORF57, has been shown to interact with both Ago2 and TNRC6, inhibit p-body formation and interfere with Ago2-miRNA targeting of mRNAs [77,78]. The mechanism of the interaction of ORF57 with RISC is somewhat different than for BGLF2, however, as ORF57 binding to Ago2 and TNRC6 disrupts the formation of the RISC complex and inhibits stress granule formation [78]. Therefore, the ability to interfere with RISC function is conserved in EBV and KSHV, although these viruses use different proteins and mechanisms to inhibit RISC activity.

In addition to the interaction of BGLF2 with RISC, the AP-MS data revealed that BGLF2 interacts with additional proteins important for RNA regulation. Notably, prominent interactions were detected with GIGYF2 (and to a less degree with GIGYF1), eIF4E2 and ZNF598, which are known to form a complex important for translation-coupled RNA decay and inhibition of translation initiation of defective mRNA [43–46]. In addition, GIGYF2 has been shown to interact with Ago2 and to possess gene silencing activity [79]. This suggests that BGLF2 might affect mRNA regulation by multiple mechanisms. The AP-MS data also identified multiple proteins which are known to localize to both stress granules and p-bodies or uniquely to stress granules, indicating that BGLF2 has multiple interactions (either direct or indirect) with stress granule components. This is in keeping with our observation that, in some cell lines, BGLF2 formed cytoplasmic bodies that localized with a stress granule marker and promoted the formation of stress granules.

To our knowledge, BGLF2 is the first EBV lytic protein identified as a miRNA regulator. There are two likely reasons why BGLF2 targets RISC. First, as we have shown here, inhibition of RISC activity provides a way to interfere with the normal function of cellular miRNAs and thereby affects the translation of cellular mRNAs. The interference with miRNA function is also suggested by the fact that BGLF2 expression led to the loss of p-bodies, which are the main sites of miRNA-mediated mRNA regulation. Secondly, since EBV encodes many miRNAs, BGLF2 may be recruiting RISC for use with EBV miRNAs. A consequence of stealing RISC for use with EBV miRNAs might then be the inhibition of cellular miRNA function, due to the unavailability of RISC for use with cellular miRNAs.

We have shown that EBV lytic infection in AGS cells is accompanied by a loss of p-bodies and shown that BGLF2 contributes to this loss. The fact that cellular p-bodies were not completely restored in our BGLF2 KO virus might involve several factors. First, our cells with the BGLF2 KO virus still produce a low level of BGLF2 (detectable at late infection times), suggesting that not all copies of the EBV episome have BGLF2 knocked out. This low level of BGLF2 could be sufficient to disrupt p-bodies. Second, since p-body numbers have been reported to double in G2 [80] and EBV lytic cells arrest in G1/S, some of the decrease in p-body numbers could be due to cell cycle effects. Thirdly, there maybe additional EBV proteins that can disrupt p-bodies that have yet to be discovered. Interestingly, it has been reported that KSHV lytic infection also leads to a loss of p-bodies [78], suggesting that disruption of p-bodies is important for γ-herpesvirus infection.

**Fig 10:**
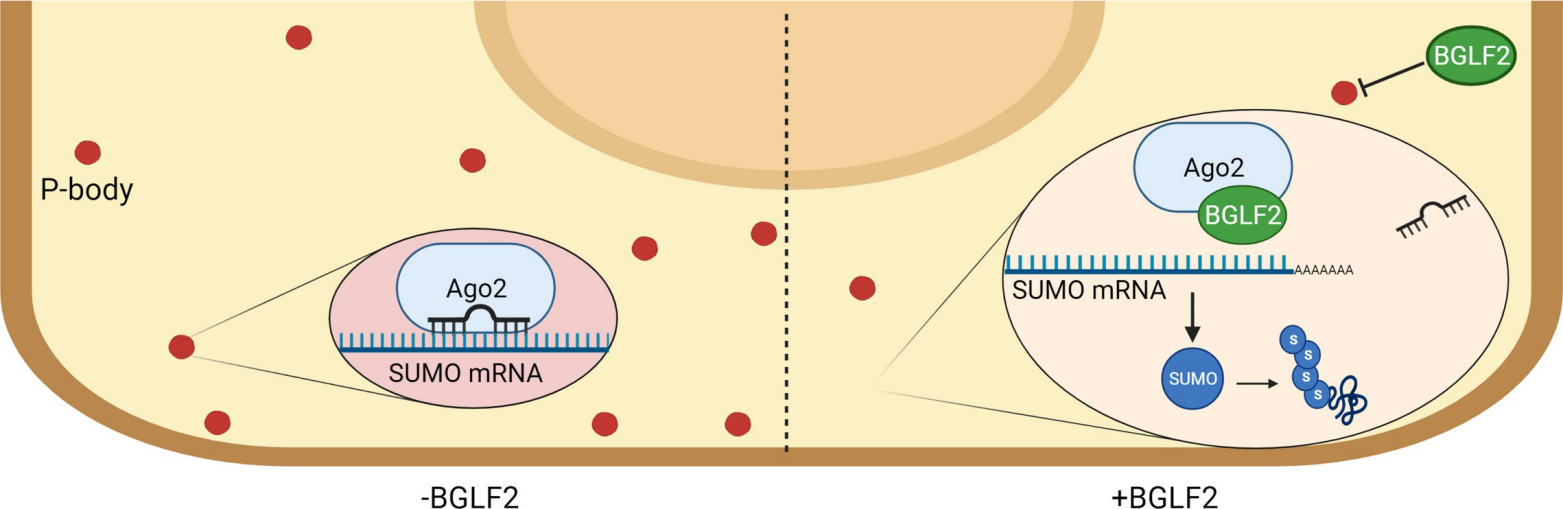
Model for BGLF2-mediated miRNA dysregulation. A model (created with BioRender.com) depicting the effect of BGLF2 on miRNA, p-bodies and SUMO. Without BGLF2 (left side), Ago2 incorporates miRNA and regulates mRNA, including SUMO transcripts, in p-bodies. When BGLF2 is present (right side), BGLF2 interacts with Ago2 inhibiting incorporation of miRNAs, including let-7 miRNAs, and decreasing the number of p-bodies. Without miRNAs incorporated, RISC can not regulate mRNA targets, including SUMO transcripts, leading to increase levels of SUMO and other encoded proteins. The increased level of free-SUMO proteins increases SUMOylation of cellular proteins; one consequence of BGLF2 expression.

We have shown that BGLF2 can regulate let-7 miRNAs function by inhibiting their incorporation with Ago2. Since BGLF2 interacts with Ago2, this inhibition may result from physical blocking of the Ago2-miRNA interaction by BGLF2, thereby interfering with the formation of p-bodies, which depend on an active miRNA silencing pathway [81] (see model in Fig 10). Since RISC is needed for the function of all cellular miRNAs, we expect that the ability of BGLF2 to inhibit miRNA function will extend to many or all cellular miRNAs. Indeed, we showed that BGLF2 also affects the ability of three additional miRNAs (miR-103a-3p, miR20a-5p and miR17-5p) to associated with Ago2, and results in the expected increase of GPRC5A protein encoded by one of the mRNA targets of miR103a-3p. Interestingly, miR20a-5p and miR17-5p have both been shown to target p21 transcripts [82], and we previously observed that BGLF2 induced p21 through post-transcriptional effects [27]. Therefore, p21 induction might be another outcome of miRNA inhibition by BGLF2. In addition, since let-7a miRNA targets Dicer transcripts, BGLF2 increases Dicer levels which might promote miRNA processing and be responsible for the increase in cellular miRNA levels we observed in cells expressing BGLF2. In summary, the impact of BGLF2 on cellular miRNA would be expected to affect many cellular processes, which might include the effects already reported for BGLF2 on the cell cycle and multiple signalling pathways.

## Materials and methods

### Cell lines

293T and HeLa cells were cultured in Dulbecco’s Modified Eagle Medium (DMEM; Multicell 319-005-CL). HONE-1, an EBV-negative nasopharyngeal carcinoma cells, were cultured in AMEM (Multicell; 310-010-CL) medium. AGS gastric carcinoma cells AGS-EBV-Z, and Raji B cells were cultured in RPMI (Multicell 350-000-CL) medium. AGS-EBV-Z cells contain the Akata strain EBV and a Tet-inducible BZLF1 gene and their generation from AGS-EBV cells [83] was previously described [22]. All media was supplemented with 10% (or 20% for Raji) fetal bovine serum (FBS; Multicell 098150) and 1% penicillin/streptomycin (Gibco 15140–122). AGS-EBV-Z cells were also supplemented with 400 μg/ml G418 (Geneticin; Gibco 10131–027) and 2 μg/ml puromycin.

AGS cells with Tet-inducible BGLF2 (AGS-BGLF2) were generated as follows: First, BGLF2-FLAG was PCR amplified from pCMV3FC-BGLF2 (using primers: 5’- TAT ACT CGA GGC CGC CAT GGC ATC CGC CGC GAA CAG TAG C-3’ (forward) and 5’- ATC GCG ACG CGT CTA CTT GTC ATC GTC ATC CTT GTA -3’ (reverse, MluI-FLAG-rev) and inserted between the Xho I and Mlu I sites in pTRIPZ-GFP [22]. This plasmid was used to generate a lentivirus in 293T cells by standard methods. AGS cells transduced with this lentivirus were grown in RPMI containing 2 μg/ml puromycin for three weeks. Individual colonies were then picked with trypsin soaked Whatman paper and propagated as for AGS cells with the addition of 1 μg/ml puromycin. To induce expression of BGLF2-FLAG, 2 μg/ml doxycycline (Sigma, D9891-SG) was added to the medium.

### BGLF2 knockout virus

The BGLF2 gene in EBV was disrupted in the context of AGS-EBV-Z cells using CRISPR/Cas9. To this end, pLentiCRISPRv2-LoxP ([84]; a gift from Reuben Harris) was modified to replace the puromycin cassette with a blasticidin (blast) cassette (pLentiCRISPRv2-LoxP-blast). The blast gene was PCR amplified from pTRIPZ-blast ([85]; a gift from Paola Scaffidi) using an N-terminal primer with a Sch I site (5’-GAC GAG TCA CGT GAT GGC CAA GCC TTT G-3’) and a C-terminal primer with a Mlu I site (5’-TAT ACG CGT TTA GCC CTC CCA CAC ATA AC-3’), then digested with Sch I and Mlu I. Since there are no unique enzymes between the original P2A and puromycin gene, P2A had to be re-inserted back into the plasmid. To this end, P2A was PCR amplified from LentiCRISRv2-LoxP using an N-terminal primer with an Nhe I site (5’-AAA GCT AGC GGC AGC GGC GC-3’) and a C-terminal primer with a Sma I site (5’-AAA CCC GGG GCC GGG GTT CTC-3’), then digested with Nhe I and Sma I. Blast and P2A digested PCR products were ligated then PCR amplified using N-terminal P2A and C-terminal Blast primers described. The P2A-Blast PCR product and LentiCRISPRv2-LoxP were digested with Nhe I and Mlu I and ligated to generate pLentiCRISPRv2-LoxP-blast.

Guide RNA targeting BGLF2 were designed using Benchling web tools (www.benchling.com/) with PAM sites of +64, predicted to cut at +61 on the antisense stand (relative to BGLF2 start codon). Guide oligos 61F 5’- CAC CGT CCT CAA CAA GGA GTG CCT C-3’ and 61R 5’-GAA CGA GGC ACT CCT TGT TGA GGA C-3’, were annealed, cut with Bsm BI, and inserted in the Bsm BI site in pLentiCRISPRv2-LoxP-blast. Lentivirus particles were generated in 293T as described above and AGS-EBV-Z cells were transduced. For AGS-EBV-Z containing non-targeting CRISPR/Cas9 for WT BGLF2 expression were generated from lentivirus containing pLentiCRISPRv2-LoxP-blast with no guide inserted. Forty-eight hours post-transduction, blasticidin (Gibco, A11139-03) and G418 were added at 10 μg/ml and 400 μg/ml, respectively, and cells were maintained under selection. For experiments involving reactivation of EBV to the lytic cycle, AGS-EBV-Z cells with BGLF2 knockout or WT BGLF2 were treated with 2 μg/ml doxycycline for 48 hours prior to harvesting the cells.

### Plasmids

EBV BGLF2, HSV-1 UL16 and CMV UL94 in pMZS3F [86] were subcloned into pCMV3FC [87] to generate proteins with C-terminal triple FLAG tags. Briefly, BGLF2, UL16 and UL94 were excised from pMZS3F with Xho I and Xba I and ligated between Xho I and Xba I sites in pCMV3FC. pCMV3FC-ORF33 was generated by subcloning KSHV ORF33 from pcDNA4-TetON-ORF33 (a kind gift from Britt Glaunsinger) between the Pst 1 and Bam HI sites of pCMV3FC. pCIneo-NHA-Ago2 [88] was a gift from Alex Palazzo. The pCIneo-λN-HA-HsTNRC6A and pCI-neo-λN-HA-HsTNRC6B [15] were gifts from Elisa Izaurralde.

Luciferase reporter plasmids pGL3 (Promega) with and without Dicer 3’UTR (kindly provided by Takashi Takahashi [58]) have been previously described, as has pRL-X [22]. pRL-let7a was generated from pRL-TK let7 A [57] by excising the TK promoter with Bgl II and Hind III, filling in the ends with Klenow and re-ligating. pEGFP-N1, pEGFP-SUMO1-3’UTR, pEGFP-SUMO2-3’UTR were gifts from Hugues de Thé and Valérie Lallemand-Breitenbach [61]. These plasmids were modified to remove the promoters and replace the GFP tag with a firefly luciferase. To this end, pEGFP-N1 and pEGFP-SUMO2-3’UTR were digested with Vsp A1 and Nhe I, and the ends were filled in with Klenow and re-ligated to generate promoterless constructs (pEGFP-X and pEGFP-SUMO2-3’UTR-X). To generate promoterless pEGFP-SUMO1-3’UTR, pEGFP-SUMO1-3’UTR and pEGFP-X were digested with Ksp A1 and Not I enzymes and the SUMO1 3’UTR fragment and digested pEGFP-X were ligated to generate pEGFP-SUMO1-3’UTR-X. The firefly luciferase gene was PCR amplified from pGL4.10 using an N-terminal primer with a Bam HI site (5’-GAT CGG ATC CGC CAC CAT GGA AGA TGC C-3’) and a C-terminal primer with a Not I site (5’-GAT CGC GGC CGC TTA CAC GGC GAT CTT GCC G-3’), then digested with Bam HI and Not I. This fragment was used to replace the Bam HI and Not I fragment of pEGFP-SUMO1-3’UTR-X, and pEGFP-SUMO2-3’UTR-X to generate pLuc-SUMO1-3’UTR-X and pLuc-SUMO2-3’UTR-X.

### Affinity purification-mass spectrometry

Three 10 cm plates of 293T cells were transfected with 5 μg of pCMV3FC-BGLF2 or pCMV3FC empty plasmid as a negative control using PolyJet (FroggaBio), as suggested by the manufacturer. Cells were transferred to 15 cm plates 24 hours later and harvested 48 hours post-transfection. Cells were lysed in 4 volumes of modified RIPA buffer (50 mM Tris-HCl pH 8, 250 mM NaCl, 0.1% sodium deoxycholate, 0.5% NP40 substitute, 2 mM EDTA) containing protease inhibitor cocktail (P8340, Invitrogen). Four milligrams of the clarified cell lysate (at 5 mg/ml) was incubated with 50 μl of M2 anti-FLAG resin (SIGMA) for 4 hours at 4°C with mixing. Resin was washed three times with RIPA buffer and proteins were eluted and trypsinized as described in Cao et al. [89]. Mass spectrometry and analysis was performed as previously described [28].

### FLAG immunoprecipitations

293T or AGS cells at ~80% confluency in 10-cm dishes were transfected with 5 μg pCMV3FC-BGLF2 using PolyJet reagent (SignaGen laboratories #52100688) as per the manufacturers protocol. Cells were expanded into 15 cm dishes after 24 hours and harvested 48 hours post-transfection. For experiments involving inducible FLAG-BGLF2, AGS-BGLF2 at ~60% confluency in 15 cm dishes were treated with 2 μg/ml Dox for 48 hours prior to harvesting. Three 15 cm plates containing AGS-EBV-Z cells at ~80% confluency were transfected with 15 μg pCMV3FC-BGLF2 (or pCMV3FC) using 30 μL PolyJet reagent. After 24 hours, cells were reactivated to lytic infection by treating with 2 μg/ml Dox for an additional 24 hours prior to harvesting. All cells were lysed on ice for 40 min in 4 volumes of modified RIPA buffer, followed by sonication and clarification by centrifugation. Clarified lysates were treated with 20 μg/mL RNase A (ThermoFisher EN0531) for 30 min at RT before being incubated with 20 μl of M2 anti-FLAG resin (Sigma-Aldrich; M8823) at 4°C for 4–16 hours with rotation. Resin was harvested by centrifugation, washed 3 times with modified RIPA buffer, and then boiled in 2x SDS loading buffer. Half of the recovered proteins and 100–300 μg input were separated on 8–12% SDS-PAGE gels and analyzed by Western blotting.

For immunoprecipitations in Raji cells, Raji cells were electroporated with pCMV3FC-BGLF2 using a Nucleofector 2b Device (Lonza) following the manufacturer’s protocol. The medium volume was doubled every 24 hours for 72 hours then cells were treated with 3 mM sodium butyrate (NaB) and 20 ng/ml 12-*O*-tetradecanoylphorbol-13-acetate (TPA) for an additional 24 hours (to reactivate EBV) prior to lysis as above. 100 μg of clarified lysate was kept for input. The remaining clarified lysate was then divided in two and incubated overnight at 4°C with IgG- or anti-FLAG-conjugated beads and 100 μl of surebeads Protein G Magnetic beads (Bio-Rad; 161–4013). Beads were collected using a magnetic rack (Bio-Rad), washed 4 times with modified RIPA buffer and then analysed by Western blotting.

For FLAG-IPs comparing BGLF2 homologues, the amount of plasmid transfected was adjusted to give equal protein expression (and made up to 5 μg with empty plasmid), and 2 mg of clarified lysates were incubated overnight at 4°C with, 5 μg rabbit anti-DDDDK (Bethyl Laboratories; A190-102A) and 50 μl of surebeads Protein A Magnetic beads preequilibrated in modified RIPA buffer. The beads were collected, washed and samples processed by Western blotting as described above.

### Western blotting

Proteins were subjected to SDS-PAGE and transferred to nitrocellulose. Membranes were blocked in 5% non-fat dry milk or 2% BSA (bovine serum albumin) in PBS-T (phosphate-buffered saline (PBS) with 0.1% Tween) and then incubated with primary antibodies: rabbit anti-DDDDK (Bethyl Laboratories; A190-102A; 1:10,000–1:40,000), mouse anti-β-actin (Santa Cruz; sc-47778, 1:5,000–1:10,000), mouse anti-vinculin (Santa Cruz; 7F9, sc-73614;1:10,000), rat anti-Ago2 (clone 11A9, Millipore Sigma; MABE253; 1:1000), rabbit anti-GW182 (TNRC6A; Bethyl, A302-329A; 1:1000), mouse anti-Dicer (Santa Cruz; A-2, sc-136981; 1:1000), mouse anti-GPRC5A (RAI3, Santa Cruz; G-6, sc-373825; 1:1000), rabbit anti-SUMO1 (Santa Cruz FL-101, 1:1000), rabbit anti-SUMO2/3 (FL-103, 1:1000), rabbit anti-SUMO1 and anti-SUMO2/3 (Abcam; ab139470; 1:5000). Primary antibodies against viral proteins were mouse anti-ZEBRA (BZLF1, Santa Cruz, sc-53904, 1:5000), rabbit anti-BGLF4 (Abgent, AP8057b; 1:1000), goat anti-vcap18 (Invitrogen, PA1-73003; 1:5000), rabbit anti-BGLF2 (a kind gift from Dr. Chien-Hui Hung; 1:1000 [29]). Membranes were washed in PBS-T, followed by incubation with secondary antibodies goat anti-mouse horseradish peroxidase (HRP) (Santa Cruz; sc-2005), donkey anti-goat (Sigma; SAB3700285), goat anti-rabbit HRP (Sigma; SAB3700878), or goat anti-rat HRP (ThermoFisher Scientific; 24555) at 1:5,000 dilution. Membranes were washed in PBS-T and developed using chemiluminescence reagents (ECL from Santa Cruz or Clarity ECL from Bio-Rad) with UltraCruz Autoradiography Film (Santa Cruz; sc-201696) or Amersham Hyperfilm ECL (Cytiva; 28906839). Protein band quantifications were done using ImageJ or Image Studio Lite (LI-COR) software.

### Effect of inducible BGLF2 on endogenous proteins and SUMOylation

AGS-BGLF2 cells were seeded in 10 cm plates at ~40–50% confluency. The next day, cells were treated with 2 μg/ml doxycycline for 24 or 48 hours to induce BGLF2-FLAG expression. Cells were harvested and lysed in 9M urea buffer (9M urea, 10 mM Tris pH 6.8), vortexed briefly and incubated for 20 min at RT. Lysates were then sonicated followed by clarification by centrifugation. Fifty to one hundred micrograms of each sample was boiled in 2x SDS loading buffer and resolved on 8% or 15% SDS-PAGE gels and analyzed by Western blotting.

### Comparison of inducible BGLF2 levels to EBV lytic infection

AGS-BGLF2 and AGS-EBV-Z cells with WT or BGLF2 KO virus were seeded in 10 cm plates at ~40–50% confluency. The next day, cells were treated with 2 μg/ml Dox for 0, 24, and 48 hours. Cells were harvested and lysates were prepared as described above. Thirty micrograms of each sample was boiled in 2x SDS loading buffer, resolved on 12% SDS-PAGE gels and analyzed by Western blotting.

### Quantitative RT-PCR for mRNA

Total RNA was isolated from cell lysates using NucleoZol (TaKaRa Bio) according to manufacturer’s instructions, and 1 μg of total RNA was suspended in 10 μl of RNase-free water. RT-qPCR was performed with 1 μl of 1:10 dilution of the RNA using Luna Universal One-Step RT-qPCR kit (New England BioLabs), with a total reaction volume of 10 μl in a Bio-Rad CFX384 Real-Time System (Bio-Rad). Primers used for Ago2 [90], Dicer [72], GPRC5A [62], SUMO3 [91] and SUMO1, SUMO2, and β-actin [23] transcripts are as previously described. The relative mRNA expression was derived from 2^-ΔΔCT^ by use of the comparative threshold cycle (CT) method. The abundance of mRNA in each sample was normalized to the amount of actin mRNA.

### Ago2 immunoprecipitation

Ago2-IP was performed as described previously [92]. Briefly, two 15 cm plates of AGS-BGLF2 cells or AGS-EBV-Z cells with WT or BGLF2 knockout virus at ~50 confluency were treated with 2 μg/ml Dox for 48 hours (or left untreated). Ten percent of the AGS-EBV-Z cells were lysed in 9M urea as protein input. The AGS-BGLF2 cells and 90% of the AGS-EBV-Z samples were then lysed in 600 μl Ago2-IP lysis buffer (25 mM Tris-HCl pH 7.4, 150 mM KCl, 2 mM EDTA, 0.5% NP-40 substitute, complete protease inhibitors) for 30 min at 4°C. Samples of the clarified lysate were removed for protein (10 μl for AGS-BGLF2) and RNA (5 μl for AGS-BGLF2 and 15 μl for AGS-EBV-Z) analyses. The remaining clarified lysate was pre-cleared by 1 hour incubation with 50 μl protein A/G magnetic beads at 4°C. Cleared lysate was then divided in two and incubated with either Ago2- or IgG_2α_-conjugated magnetic beads overnight at 4°C with rotation. Conjugated beads consisted of 100 μg protein A/G magnetic beads (GB-Magnetic; 2220213) coupled to 1 μg rat anti-Ago2 (clone 11A9, Millipore; MABE253) or rat IgG_2a_ (Millipore; MABF1077Z). Beads were washed 3 times with Ago2-IP washing buffer (50mM Tris-HCl pH 7.4, 300 mM KCl, 1 mM MgCl2, 0.5% NP-40 substitute) then resuspended in 500 μl PBS. 25 μl of each sample in PBS was removed for Western blot to quantify Ago2 recovery. The remaining beads and 5 μl of cleared lysate were suspended in 1 ml NucleoZol for RNA extraction and recovered mRNAs and miRNAs were quantified as described above and below, respectively. Percent input and fold-enrichment was adjusted to relative recovery of the Ago2 protein in each IP, as determined by Western blotting for Ago2 and quantification using ImageJ software.

### Quantitative RT-PCR for miRNA

miRNA cDNA and real-time PCR was performed on total RNA following the miRCURY LNA miRNA PCR kit (Qiagen) protocol. Five nanograms of UniSp6 (spike-in control) was added to each RNA sample before generating cDNA. Primers targeting specific miRNAs are as follows: hsa-let-7a-5p (YP00205727), hsa-let-7g-5p (YP00204565), hsa-miR-103a-3p (supplied with PCR kit), hsa-miR-20a-5p (YP00204292), hsa-miR-17-5p (YP02119304), and UniSp6 (supplied with PCR kit) and U6 (YP00203907). All Cq values were normalized to UniSp6 Cq to account for cDNA amplification. For inputs, the abundance of miRNAs in each sample was normalized to the amount of U6.

### Luciferase reporter assays

HONE-1 cells were plated in a 6 well plate at ~2 x 10^5^ per well. 24 hours later, cells were co-transfected with 0.5 μg firefly luciferase reporter plasmids pGL3 or pGL3-Dicer3’UTR, 0.2 μg pRL-X (an internal control for nonspecific expression effects) and 3.3 μg of either pCMV3FC-BGLF2 or pCMV3FC (empty plasmid control) using Lipofectamine 2000. As a positive control, mSCV-let-7-sponge (Addgene plasmid 29766) or mSCV empty plasmid control (received from Lin He [93]), were co-transfected with the firefly and *Renilla* luciferase constructs. To measure effects of let-7a regulation, the reporters were replaced by pRL-X and pRL-let-7a-X (containing two tandem *let-7a* binding sites) and the internal control was replaced by pGL4.10 (Promega). To measure effects of SUMO 3’UTRs, the luciferase reporter plasmids were replaced by pGL4.10, pLuc-SUMO1-3’UTR-X, or pLuc-SUMO2-3’UTR-X (using pRL-X as the nonspecific control). Luciferase assays were performed 24 hours following transfection using a dual-luciferase reporter assay system (Promega) according to manufacturer’s protocol. Respective luciferase activity (firefly or *Renilla*) was normalized to internal control luciferase activity for each sample and the average values from three independent experiments were normalized relative to the value for an empty plasmid control.

### Immunofluorescence microscopy

HONE-1 and HeLa cells were transfected with 1 μg pCIneo-λN-HA-HsTNRC6A, pCIneo-λN-HA-HsTNRC6B, or pCIneo-NHA-Ago2 with 1 μg pCMV3FC-BGLF2 or pCMV3FC using Lipofectamine 2000 (Invitrogen, 52887) at a 1 μg:2 μl DNA:Lipofectamine ratio. 24 hours post-transfection, HONE-1 cells were fixed as previously described [22]. 24 hours post-transfection, HeLa cells were trypsinized and re-seeded equally into two wells of a 6-well plate containing polylysine treated coverslips. After an additional 24 hours, HeLa cells were fixed as previously described [22]. AGS-BGLF2 and AGS-EBV-Z WT and BGLF2 KO cells were seeded into 6-wells containing polylysine treated coverslips. After 24 hours, cells were treated with 2 μg/ml Dox for 24 hours or left untreated, then fixed as previously described [22]. Coverslips were incubated with primary antibodies against FLAG (M2 mouse, Sigma-Aldrich; F1084; 1:1000), HA (rabbit, Cell Signaling, 37245; 1:1000), Ago2 clone 11A9 (rat, Millipore Sigma, MABE253; 1:100), GW182 (TNRC6A; Bethyl, A302-329A; 1:100), Dcp1a (mouse, Santa Cruz, sc-100706; 1:100), G3BP1 (rabbit, Cell Signaling, 177985; 1:100). Primary antibodies were detected using goat anti-rat Alexa Fluor 488, or goat anti-mouse or goat anti-rabbit Alexa Fluor 555 or 647 secondary antibodies (Invitrogen) at a 1:700 dilution. Coverslips were mounted onto slides and visualized as previously described [22].

### Analysis of stress granules

HeLa cells were seeded and transfected with as described above. After 24 hours, cells were left untreated or treated with the following compounds: 20 μg/ml puromycin, 10 μg/ml emetine, 10 μg/ml cycloheximide for 1 hour or 0.5 mM sodium arsenite for 30 minutes prior to fixing as described above. Coverslips were incubated with primary antibodies against M2 FLAG and rabbit G3BP1. Primary antibodies were detected using goat anti-mouse Alexa Fluor 647 or goat anti-mouse Alexa Fluor 555 secondary antibodies at a 1:700 dilution. Coverslips were mounted onto slides and visualized as previously described [22]. Fifty cells each of FLAG negative and FLAG positive cells were counted for presence of G3BP1 foci, indicative of stress granules.

## Supporting information

S1 Fig Lack of stress granules in AGS cells and effect of BGLF2 on p-bodies in HONE-1 cells. (A) AGS cells were treated with 20 μg/ml puromycin for 1 hour or left untreated prior to being fixed and stained with DAPI and antibodies against G3BP1. Scale bar = 10 μm (B) HONE-1 cells were transfected with pCMV3FC-BGLF2 prior to being fixed and stained with DAPI and antibodies against FLAG and Dcp1a. P-bodies were counted in 50 FLAG negative (FLAG-ve) and 50 FLAG positive (FLAG+ve) cells. ** = 0.001<P≤0.01. Scale bar = 25 μm.
(TIF)

S2 Fig Ago2-IP in AGS-BGLF2 and AGS-EBV-Z WT and BGLF2 KO cells. (A) Ago2 was immunoprecipitated from AGS-BGLF2 cells treated (+) or untreated (-) with Dox using anti-Ago2 antibody or IgG negative control antibody as described in Fig 8A. Five percent of the input lystate and 1% of the IP were analysed by Western blotting using antibodies against Ago2 and FLAG. (B) Ago2 was immunoprecipitated from AGS-EBV-Z cells containing WT or BGLF2 KO virus as described in Fig 8D. Five percent of the input lysates and 0.8% of the IPs were analysed by Western blotting using anti-Ago2 antibody.
(TIF)
