# Supplementary figures and images for: Epstein-Barr Virus BGLF2 commandeers RISC to interfere with cellular miRNA function
Source: PLoS Pathog. 2022 Jan 10;18(1):e1010235. doi: 10.1371/journal.ppat.1010235 (PMC8782528; doi:10.1371/journal.ppat.1010235)

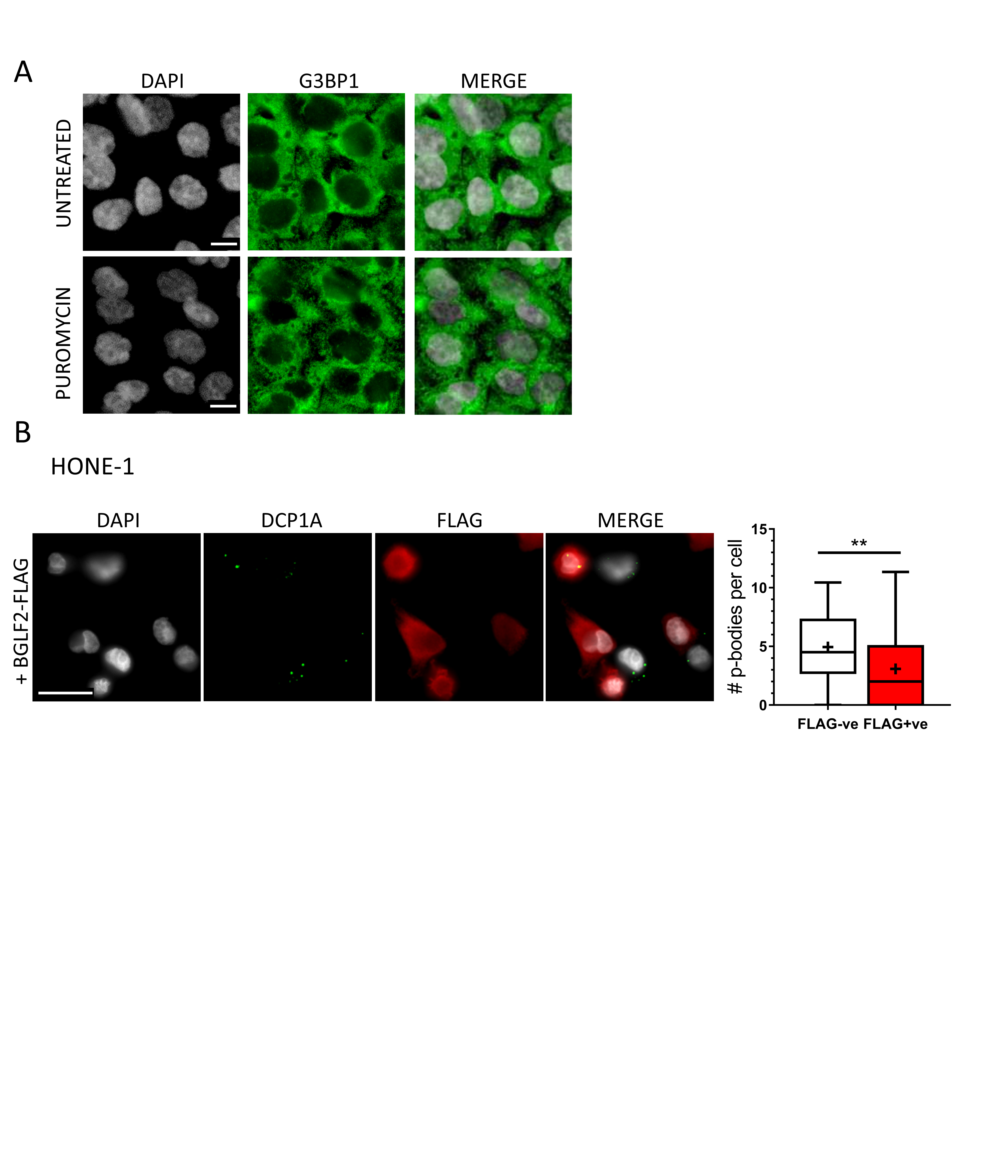

Supplement: S1 Fig — (A) AGS cells were treated with 20 μg/ml puromycin for 1 hour or left untreated prior to being fixed and stained with DAPI and antibodies against G3BP1. Scale bar = 10 μm (B) HONE-1 cells were transfected with pCMV3FC-BGLF2 prior to being fixed and stained with DAPI and antibodies against FLAG and Dcp1a. P-bodies were counted in 50 FLAG negative (FLAG-ve) and 50 FLAG positive (FLAG+ve) cells. ** = 0.001<P≤0.01. Scale bar = 25 μm. (TIF) [file ppat.1010235.s001.tif]

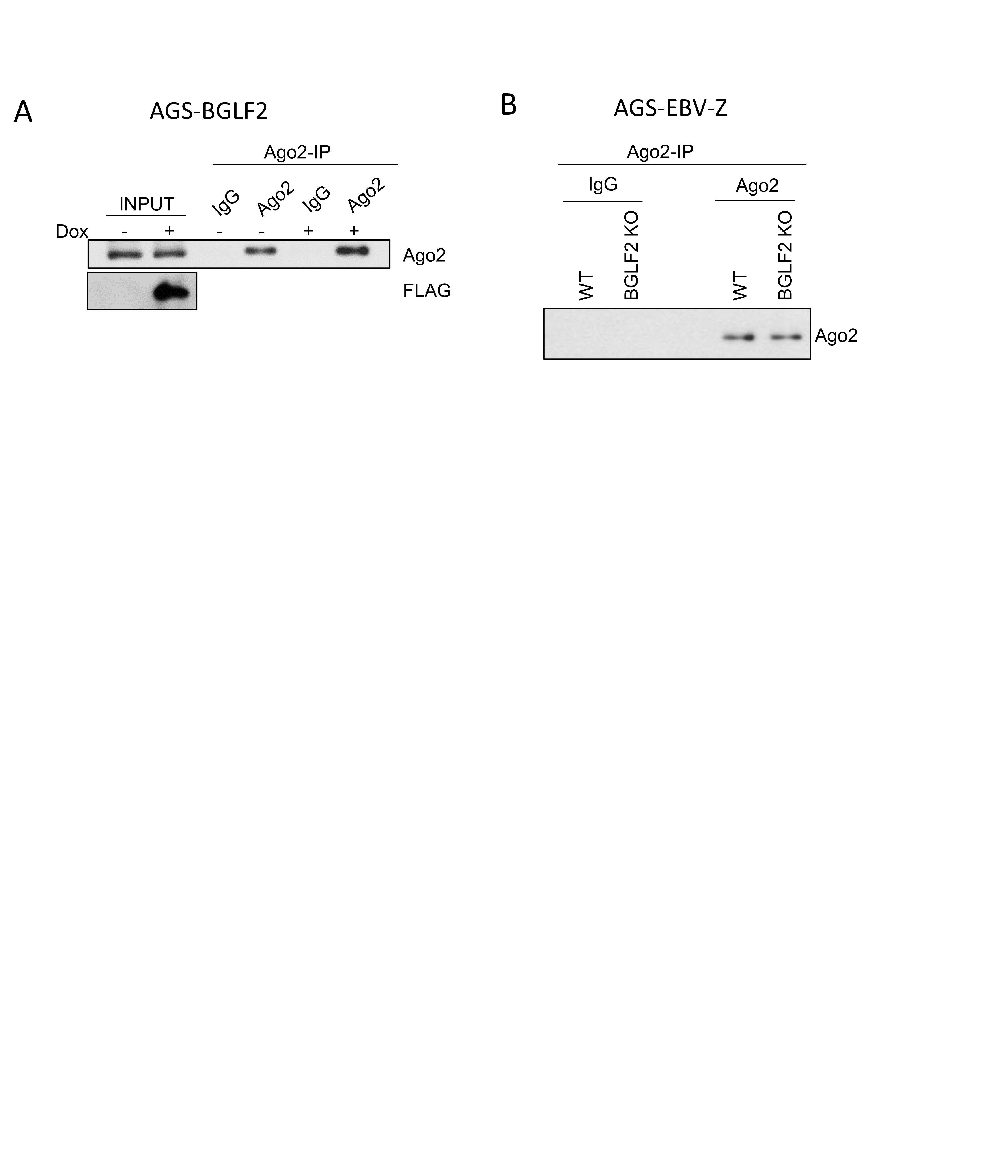

Supplement: S2 Fig — (A) Ago2 was immunoprecipitated from AGS-BGLF2 cells treated (+) or untreated (-) with Dox using anti-Ago2 antibody or IgG negative control antibody as described in Fig 8A. Five percent of the input lystate and 1% of the IP were analysed by Western blotting using antibodies against Ago2 and FLAG. (B) Ago2 was immunoprecipitated from AGS-EBV-Z cells containing WT or BGLF2 KO virus as described in Fig 8D. Five percent of the input lysates and 0.8% of the IPs were analysed by Western blotting using anti-Ago2 antibody. (TIF) [file ppat.1010235.s002.tif]
